# Mitochondrial DNA Consensus Calling and Quality Filtering for Constructing Ancient Human Mitogenomes: Comparison of Two Widely Applied Methods

**DOI:** 10.3390/ijms23094651

**Published:** 2022-04-22

**Authors:** Alexandros Heraclides, Eva Fernández-Domínguez

**Affiliations:** 1 Department of Health Sciences, European University Cyprus, Diogenis Str. 6, Nicosia 2404, Cyprus; 2 Department of Archaeology, Durham University, South Road, Durham DH1 3LE, UK; eva.fernandez@durham.ac.uk

**Keywords:** archaeogenetics, mtDNA, consensus sequence calling

## Abstract

Retrieving high-quality endogenous ancient DNA (aDNA) poses several challenges, including low molecular copy number, high rates of fragmentation, damage at read termini, and potential presence of exogenous contaminant DNA. All these factors complicate a reliable reconstruction of consensus aDNA sequences in reads from high-throughput sequencing platforms. Here, we report findings from a thorough evaluation of two alternative tools (ANGSD and schmutzi) aimed at overcoming these issues and constructing high-quality ancient mitogenomes. Raw genomic data (BAM/FASTQ) from a total of 17 previously published whole ancient human genomes ranging from the 14th to the 7th millennium BCE were retrieved and mitochondrial consensus sequences were reconstructed using different quality filters, with their accuracy measured and compared. Moreover, the influence of different sequence parameters (number of reads, sequenced bases, mean coverage, and rate of deamination and contamination) as predictors of derived sequence quality was evaluated. Complete mitogenomes were successfully reconstructed for all ancient samples, and for the majority of them, filtering substantially improved mtDNA consensus calling and haplogroup prediction. Overall, the schmutzi pipeline, which estimates and takes into consideration exogenous contamination, appeared to have the edge over the much faster and user-friendly alternative method (ANGSD) in moderate to high coverage samples (>1,000,000 reads). ANGSD, however, through its read termini trimming filter, showed better capabilities in calling the consensus sequence from low-quality samples. Among all the predictors of overall sample quality examined, the strongest correlation was found for the available number of sequence reads and bases. In the process, we report a previously unassigned haplogroup (U3b) for an Early Chalcolithic individual from Southern Anatolia/Northern Levant.

## 1. Introduction

Since its emergence in the 1990s [1], the field of human archaeogenetics has seen an unprecedented expansion, moving from mitochondrial DNA (mtDNA) analysis of just a handful of ancient samples in the early 2000s [2,3,4], to the reporting of whole-genome data for hundreds of ancient individuals at once [5,6] aided by the advent of high-throughput sequencing (HTS) techniques. The wealth of information provided by the analysis of whole-genome ancient DNA (aDNA), and the evident revolution it brought in anthropology, archaeology, and history [7,8], resulted in a relative lack of interest in comprehensive, systematic, accurate, and standardized analysis of uniparental markers, particularly mtDNA, in ancient samples. However, phylogenetic and phylogeographical analysis of ancient mtDNA can reveal deep matrilineal ancestry, enriching findings from autosomal DNA and providing a complete picture of human evolution and past migrations [9,10]. In particular, mtDNA analysis helped reveal for the first time the genetic discordance between early Neolithic European farmers and their contemporary hunter–gatherers [11] and provided unequivocal evidence for a demic diffusion of agriculture via a massive migration of Near Eastern farmers into Europe [12,13]. More recently, ancient mtDNA revealed a rapid single dispersal of all non-Africans fewer than 55,000 years ago and a previously unknown major population shift in Europe at the end of the Pleistocene [14,15]. Additionally, extensive pedigrees can be reconstructed using ancient mtDNA phylogeny, which could help elucidate archeologically and anthropologically interesting issues on nuclear and extended families, kinship, and social connections in the ancient world [16]. A recent example of such an approach, combining information from both autosomal and uniparental ancient DNA analysis, revealed a high-resolution picture of extended family dynamics and sophisticated kinship burial practices in Neolithic Britain [17].

Another advantage of analyzing mtDNA from ancient samples is that due to its higher number of cellular copies, it is more readily available and easier to extract and amplify in sufficient amounts compared with nuclear DNA [9]. This is particularly important for ancient samples due to degradation of nucleic acid molecules occurring *post-mortem* through time, exacerbated by environmental factors such as high temperature, high humidity, and salinity and extreme pH [18]. More specifically, endogenous aDNA shows typical signs of damage, particularly at read termini, resulting from hydrolysis and oxidation that act on the biological sample through time. While fragmentation primarily results from depurination, miscoding lesions stem from deamination of cytosines in forward and reverse DNA strands. After sequencing, this may appear as C>T misincorporations at 5′ and G>A at 3′ ends [19]. An additional major issue faced in aDNA analysis is contamination through the presence of exogenous modern DNA [20].

Due to its higher abundance, mtDNA may be potentially less prone to all the above processes even in low-coverage and/or highly degraded genetic samples. Still, accurately reconstructing mitochondrial sequences from ancient samples poses several challenges, since false positive base calls at crucial positions, resulting from fragmented, damaged, or contaminated aDNA, might compromise the accuracy of the derived haplotype, resulting in erroneous haplogroup predictions and biased downstream analysis. Other intrinsic characteristics of mtDNA polymorphic regions, such as the presence of poly-C tracts in hypervariable regions I and II, can result in misalignments and incorrect base calling and reporting when length heteroplasmy occurs [21,22].

Given the above, the accuracy of mitogenome reconstruction from raw genomic data is of paramount importance. Several tools and relevant pipelines have been developed for dealing with DNA degradation [23,24,25], as well as contamination [26,27], but just a few have been actually implemented to reliably reconstruct endogenous mitogenome consensus sequences [24,28].

Here, we report findings from a thorough evaluation of two alternative tools and pipelines, namely schmutzi [24] and ANGSD [28], aimed at detecting and reducing the impact of damage and contamination over ancient DNA fragments, and reconstruct, wherever possible, high-quality ancient mitogenomes from publicly available raw genomic data.

## 2. Results

Characteristics of the 17 previously published ancient human samples [29,30] analyzed in the current study can be found in Table 1 (more detailed characteristics in Table S1). The 17 ancient individuals were identified in archeological sites from the ancient Near East (Anatolia, Levant, and the Caucasus) and were previously dated from the 14th to the 7th millennium BCE (from the Epipaleolithic to the Chalcolithic era). Given their ancient nature, these samples ranged from moderate coverage (e.g., ZBC 2.9×) to low coverage (e.g., ZKO 0.90×) to very low coverage (e.g., KFH2 0.16×). In fact, 88% (15 to 17) of samples had a mean coverage < 1×. The proportion of damage/deamination at 5′ terminal position was relatively high (>0.20) for 53% (9 out of 17) samples, while exogenous contamination as reported in the original studies was low (<5%).

### 2.1. Derived Mitogenome Consensus Sequences

#### 2.1.1. Schmutzi Pipeline

Schmutzi [24], with its sophisticated pipeline, is generally considered the gold standard in regard to identifying deamination patterns (used as markers for presence of endogenous aDNA) and estimation of exogenous mitochondrial contamination, simultaneously calling the mitochondrial consensus sequence. Here, analyzing publicly available raw genomic data (FASTQ format), including very low-quality, previously not mitochondrially analyzed samples, we managed to reconstruct the mitogenome sequence of 16 out of 17 samples analyzed. As expected, the derived mitogenomes had gaps in their sequence with non-called bases (usually represented with an “N” instead of a base in the sequence). Given the high fragmentation and damage characterizing aDNA, such gaps are anticipated and generally considered acceptable, as long as they do not compromise mtDNA haplogroup prediction and/or downstream haplotype analysis. The single mitogenome that could not be reconstructed using this method, belonged to a very low-quality sample (KRD004) [30], characterized by very low overall and mitochondrial (MT) coverage (0.06× and 0.5×, respectively), very low number of reads mapping the mtDNA and total base calls (173 and 8551, respectively), as well as high proportion of damage/deamination at the 5′ terminal position on the MT (0.32). The determining factor for the application not running is apparently the very low number of aDNA fragments, which prevents the estimation of misincorporation patterns due to deamination, as indicated by the program developer [24].

#### 2.1.2. ANGSD Pipeline

ANGSD [28], through its “-*dofasta*” option, creates a FASTA consensus sequence from a raw genomic data file (BAM format). After isolating the MT chromosome from the whole-genome raw data (wherever required), we reconstructed the mitogenome sequence of all 17 analyzed samples, including the very low-quality sample (KRD004) not called by schmutzi, as described above. As in the case of schmutzi-derived sequences, those derived by ANGSD had gaps due to non-called bases. It should be noted that none of the two publications originally reporting these samples [29,30] used this approach for reconstructing the mitochondrial consensus sequence.

### 2.2. Quality-Improved Mitogenome Sequences through Filtering 

#### 2.2.1. Schmutzi Filtering

By using sequence deamination patterns and fragment length distributions, the default schmutzi pipeline, even before any post-reconstruction quality filtering, is anticipated to accurately call the endogenous mitochondrial sequence [24]. Schmutzi’s “log2fasta” program has been designed to apply quality cut-offs to further improve the quality of the initially derived mitochondrial consensus sequence by filtering out low quality bases. By testing a series of consecutive cut-offs (Q5, Q10, Q15, Q20, Q25, and Q30), we could reconstruct consensus sequences for 11 out of the 17 analyzed samples (Table S2). For 7 out of 11 samples, there was a substantial improvement in the sequence quality, as indicated by a marked improvement in haplogroup prediction quality and particularly a reduction in the estimated erroneous base calls (discussed in the following subsection). Some high-coverage samples, in which none or very low ambiguous calls were obtained pre-filtering (e.g., CBT018, MTT001), did not show any marked improvements, as expected, due to a ceiling effect (i.e., the prediction quality has a limit of 1, thus any prediction quality initially close to that does not have much room for improvement). It should be noted that the improvement by basequality filtering was not observed in a dose–response manner (i.e., higher/stricter cut-offs do not necessarily lead to better quality sequences).

The six samples (BAJ001, KRD001. KRD002, KRD004. KRD005, and KRD006) for which filtering failed to construct mitogenome consensus sequences were all low coverage (0.75×, 0.20×, 0.15×, 0.06×, 0.03×, and 0.21×, respectively), with a relatively low number of total and MT reads and base count (17,270; 10,953; 14,643; 8551; 28,877; 52,582) and relatively high damage at the MT 5′ terminal (0.28; 0.22; 0.27; 0.21; 0.18; 0.19; respectively).

#### 2.2.2. ANGSD Filtering

Unlike schmutzi, ANGSD and its “-*dofasta”* option have not been explicitly designed to analyze ancient mitogenomes and, more specifically, to deal with the special characteristics of such samples (e.g., deamination and contamination). Given this, it is essential that filters are applied when reconstructing ancient mitogenomes from raw data. Two relevant filters applied in our analyses are: (i) trimming bases at both ends of reads (particularly useful for aDNA analysis) and (ii) using cut-offs on base quality (comparable to schmutzi’s filter). This trimming (1, 3, and 5 bases) and base quality (Q5, Q10, Q15, Q20, Q25, and Q30) filtering resulted in the consensus sequence reconstruction of all 17 analyzed samples (Table S2). Interestingly, the stricter quality pipeline of schmutzi, could only reconstruct 11 consensus sequences postfiltering. As in the case of schmutzi, the postfiltering consensus sequences derived by ANGSD were generally of better quality than the prefiltering sequences (discussed in the following subsections).

### 2.3. Mt Haplogroup Determination and Evaluation of Overall Sample Quality 

Mitochondrial haplogroups were determined by HaploGrep 2 [31] using the sequences derived by both tools (schmutzi and ANGSD) for all 17 samples (Table 1 and Table S1). It should be noted that for six of these samples (KRD001, KRD002, KRD003, KRD004, KRD005, and KRD006) no haplogroup was reported in the original study given the very low coverage and scarcity of MT reads. In the current analysis, there was concordance as regards the predicted haplogroup (as reported in the corresponding original study) for all ANGSD-derived mitogenome sequences (11 out of 11), while for the schmutzi-derived sequences and the haplogroup of three samples was either not predicted at all due to complete failure to analyze the raw data (sample KRD004), or was predicted with very low accuracy due to failure to construct the consensus sequence postfiltering (samples BAJ001 and KRD002). It should be noted that all three samples were of very low coverage (0.75×, 0.15×, and 0.06×, respectively), with a very low number of MT reads (340; 338; 173, respectively) and a relatively high proportion of damage (0.28; 0.27; 0.21; respectively).

**Figure 1:**
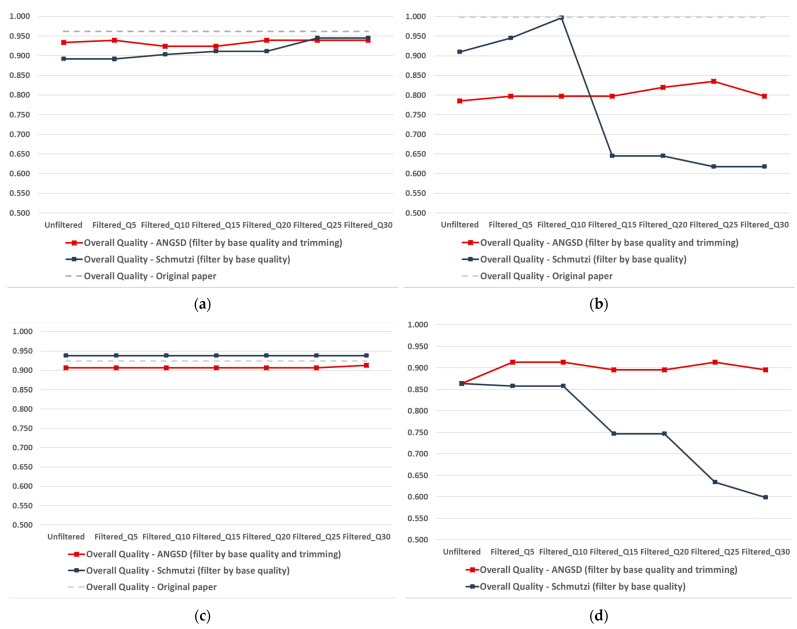
Haplogroup prediction accuracy using mitogenome consensus sequence extracted from raw genomic data, as derived by two alternative tools and pipelines (ANGSD vs. schmutzi). Selected samples shown (display of all samples in Figure S1): (**a**) ZBC [29] is a moderate-coverage sample, in which quality filtering slightly improved overall quality; (**b**) ZMOJ [29] is a low coverage sample, in which quality filtering had a differential effect on overall quality, as applied by the two alternative tools; (**c**) ZHJ [29] is a low-coverage sample, in which quality filtering did not have any effect on the overall quality; (**d**) KRD003 [30] is a very low-coverage sample, in which quality filtering had a differential effect as applied by the two alternative tools, with a progressive reduction in the overall quality for one tool.

The derived HaploGrep’s quality score (range from 0 to 1) provides an indication of the quality of the input sequence, based on observed and expected polymorphisms for the predicted haplogroup, as well as the remaining polymorphisms in the sample. Sample qualities for the unfiltered and filtered (by base quality in schmutzi and by trimming plus base quality in ANGSD) for all analyzed samples can be seen in Figure S1 (selected samples of interest are displayed in Figure 1). The ANGSD and schmutzi quality scores were plotted against the original paper quality score for each sample, which acted as the gold standard. It should be noted that, for the ANGSD filtering, Figure 1 and Figure S1 only display the best trimming choice (1, 3, or 5 bases) in combination with a series of base-quality filters for each sample. The improvement in quality score for each ANGSD trimming option can be found in Table S2.

As noted previously, the improvement in the quality score postfiltering was apparent for both tools, with the maximum score reached at different filters for each sample and each tool. For some samples, the maximum quality score was achieved before reaching the most stringent filtering cut-off (i.e., Q30) and, in fact, additional stringency in filtering resulted in worse overall HaploGrep quality scores for some samples (e.g., ZMOJ, ZKO, ZHAJ, and KRD003), particularly when running schmutzi’s log2fasta. For very few samples (e.g., CBT018 and MTT001), filtering did not improve the quality scores at all, but it should be noted that these samples achieved very high HaploGrep quality scores even in the unfiltered sequence, reflecting high-quality samples. Overall, schmutzi’s filtering appeared to result in a more pronounced change in the sample quality score compared with ANGSD’s filtering.

**Figure 2:**
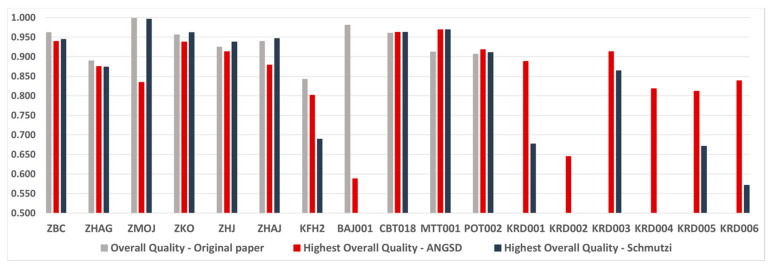
Highest haplogroup prediction accuracy for all samples using mitogenome consensus sequence extracted from raw genomic data by two alternative tools and pipelines (ANGSD vs. schmutzi). Absence of a bar for one of the two tools indicates that either there was a wrong haplogroup perdition or that no consensus sequence could be derived. Absence of a bar for the original paper indicates that no haplogroup was predicted in the original study due to the poor sequence quality.

Generally, the quality scores achieved through the current analysis were comparable to those achieved in the original studies, where the specific samples were first reported [29,30], with the exception of two samples (KFH2 and BAJ001) (Figure 2). Of note is that for a few samples (five in the case of schmutzi and three in the case of ANGSD), the mitogenome sequences reconstructed in the current analysis resulted in higher haplogroup quality scores compared with those achieved in the corresponding original studies (Figure S2).

**Figure 3:**
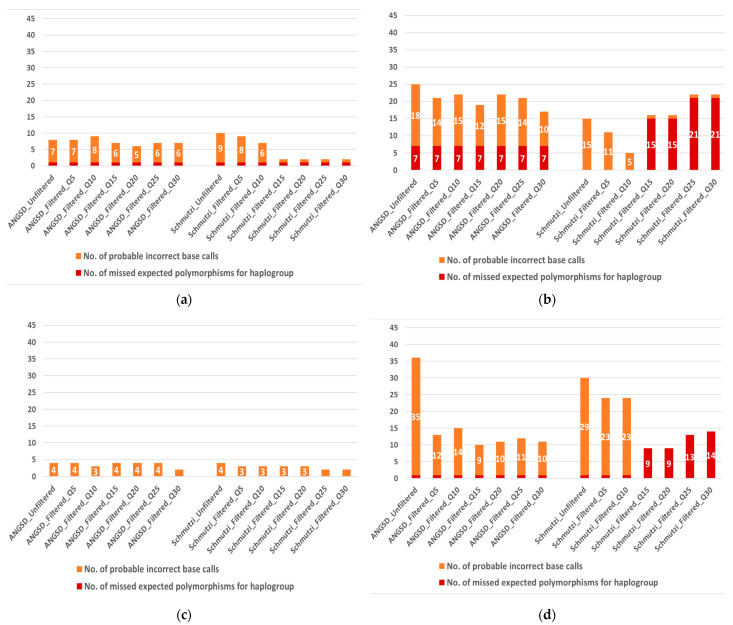
Possible errors in mitochondrial consensus sequence calling for analyzed samples as indicated during haplogroup prediction, presented by base-quality filter, by two alternative tools, and pipelines (ANGSD vs. schmutzi). Selected samples shown (display of all samples in Figure S2): (**a**) ZBC [29] is a moderate-coverage sample, in which quality filtering had a slightly differential effect on sequence calling errors, as applied by the two alternative tools; (**b**) ZMOJ [29] is a low-coverage sample, in which quality filtering had a clear differential effect on sequence calling errors, as applied by the two alternative tools; (**c**) ZHJ [29] is a low-coverage sample, in which quality filtering did not have any substantial effect on sequence calling errors; (**d**) KRD003 [30] is a very low-coverage sample, in which quality filtering had a clear differential effect on sequence calling errors, as applied by the two alternative tools.

Additional to the overall quality score, HaploGrep provides errors and warnings, following each haplogroup prediction based on a given input mitogenome sequence, namely missed expected polymorphisms for the predicted haplogroup and presence of global private mutations not reported in the current mitochondrial Phylotree [32]. These are presented in Figure S2 for all analyzed samples (selected samples of interest are displayed in Figure 3). It should be noted that the number of detected errors appears to be proportional to the overall quality (i.e., the higher the number of errors the lower the overall quality score) (Table S3). As in the case of the overall quality (Figure 1 and Figure S1), the number of errors decreased gradually by applying cut-offs of increasing quality filters. Unlike the overall quality score, for most samples this reduction in errors was observed in a dose–response manner (the stricter the cut-off the lower the number of errors), resulting from the filtering out of false positive base calls. The main reason that more stringent filtering did not always result in better overall sample quality (Figure 1 and Figure S1) has to do with the fact that along with false-positive base calls, filtering tends to remove true-positive base calls, particularly if the quality of the sample is not high, thus haplogroup prediction might be compromised.

It should be noted that for some samples it was not possible to estimate the number of sequence call errors, either due to failure of calling the consensus sequence altogether (either pre- or post-filtering), or due to the wrong haplogroup determination. This was particularly apparent with schmutzi, whose stricter filtering tended to remove all bases from the sequence for low-quality samples (e.g., samples BAJ001, KRD001, and others.).

### 2.4. Predictors of Sample Quality Score 

**Figure 4:**
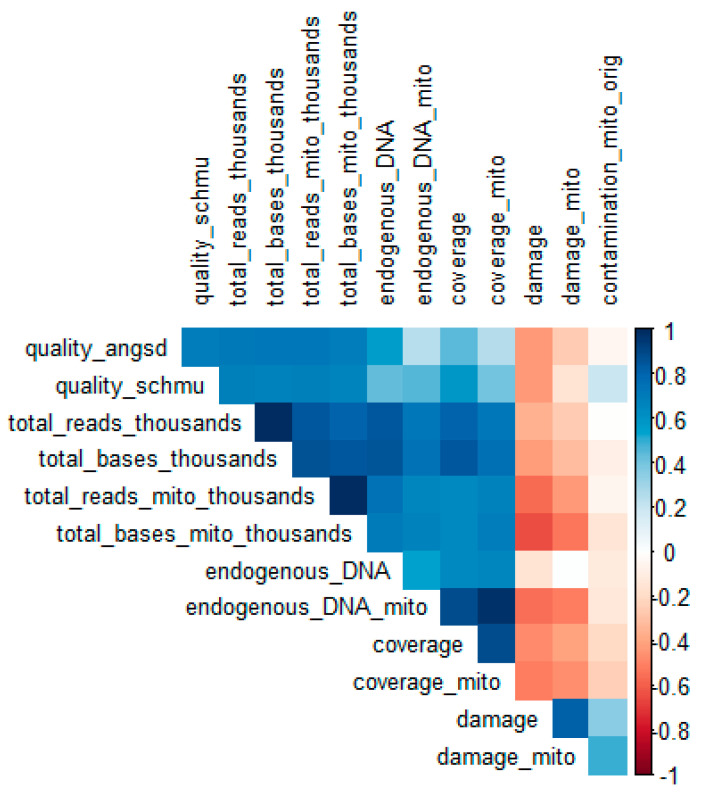
Correlation matrix displaying pairwise correlation coefficients between all sample characteristics tested, including the highest overall sample quality achieved during haplogroup prediction, for the newly derived consensus sequences, using the two alternative tools (ANGSD vs. schmutzi). Blue color indicates positive correlation and red color negative correlations. Positive and negative correlation coefficients, on the right-hand side of the plot, similarly indicate the strength of associations. The deeper the color the stronger the correlation. Scatter plots for the strongest correlations between predictors and overall sample quality can be found in Figure S3.

In an attempt to detect predictors of haplogroup quality score, a correlation matrix was constructed between all sample characteristics, including highest overall prediction quality for each sample for both tools (Figure 4). Furthermore, the best-quality score achieved for each sample with both tools was used as the outcome in a linear regression model, including different sequencing parameters, in turn, as independent variables (Figure S3 and Table S3). This analysis revealed that the whole genome (1240 K capture) sequencing data were somewhat more predictive of the derived sequence-quality score than the data from the mitogenomes (mitocapture). Overall, the strongest predictor of the quality score for both tools were the total number of reads and bases (both in the whole genome and specifically on the MT) and to a lesser extent the overall coverage (Table S3).

As expected, the extent of deamination/damage at 5′ terminal position was inversely correlated with the derived sequence quality score for both tools, while the extent of exogenous contamination (as estimated in the original studies) was not associated with the quality score. It should be noted here, however, that contamination as estimated in the original studies was particularly low for all samples, since this is a criterion for selecting them for formal analysis in the original publications.

## 3. Discussion

### 3.1. Reconstructing Ancient Mitogenomes from Raw Genomic Data 

Analyzing raw genomic data (FASTQ/BAM format) from 17 previously published whole-genome ancient samples ranging from the 14th to the 7th millennium BC, we have effectively reconstructed complete mitogenome sequences, using two widely used tools (schmutzi and ANGSD). These samples include six newly derived mitogenomes not previously analyzed, which are generally of very low coverage and acted as a good indicator of the limits of the two tools used.

The main finding from our analysis on the initial reconstruction of mitogenomes from the aforementioned samples is that the coverage of the raw genomic sample and, in particular, the available number of sequenced reads and bases (both total and on the MT), have a major determining role on the success and accuracy of ancient mitogenome reconstruction. From the two tools used, even though schmutzi was explicitly designed to handle ancient mtDNA genomic data, it is limited greatly by the above factors, to the extent of not being able to run in extreme cases (i.e., number of reads on MT < 200 and/or number of bases on MT < 10,000), such as sample KRD004 [30] reanalyzed in our study. This is apparently the reason that such samples are not mitochondrially analyzed in published studies focusing on whole-genome data, since schmutzi is the tool of choice in the majority of these, e.g., [33,34,35].

On the contrary, ANGSD’s “*dofasta*” option is able to call the mitochondrial consensus sequence even in very low-coverage samples, albeit with questionable accuracy. This accuracy can, however, be easily determined following the procedure reported in the current study (further discussion on this in the following subsection). Even though the use of schmutzi, or any other available tool estimating mtDNA contamination and automatically calling the consensus sequence, appears to be an attractive and convenient approach, we recommend at least attempting to derive the consensus sequence with other available tools (e.g., ANGSD in our case) when schmutzi is unable to run due to a low number of available sequence reads/bases.

### 3.2. Improving the Quality of Reconstructed Ancient Mitogenomes through Filtering 

Quality-filtering of raw sequence aDNA data serves the purpose of reducing the possibility of false positive base calls, resulting either from low number of reads, damage/deamination, or exogenous contamination. Our analysis revealed that, for both tested tools (schmutzi and ANGSD), quality filtering fulfills this purpose, with schmutzi, however, failing to provide filtered consensus sequences for very low-coverage samples (BAJ001, KRD001, KRD002, KRD005, and KRD006) as a result of filtering out all bases in the sequence, even with the lowest base quality score cut-off used (Q5), apparently due to the overall low quality of these samples. As in the case of prefiltering consensus sequence calling, ANGSD was able to reconstruct filtered consensus sequences for all tested samples (Figure S1, Table S2).

Despite the above, schmutzi’s quality filtering, based on the base q score, was particularly successful in improving the quality of the consensus sequence in low- to moderate-coverage samples by effectively filtering out false-positive base calls (Figure 1 and Figure S1, Table S2). It should be noted, however, that increasing stringency in the quality cut-off used (e.g., Q5, Q10, Q15, Q20, etc.) does not necessarily improve the quality in a ‘dose–response’ manner for all samples. Given this, we strongly recommend not relying on predefined cut-offs for filtering out low-quality bases (e.g., Q20 or Q30 for all samples), since this (as clearly indicated with the current findings) can bias the derived consensus sequence for some samples. To provide a specific example for this phenomenon using the current findings, if a base quality cut-off of say Q30 was applied to all analyzed samples, this would result in a consensus sequence of lower accuracy (lower-quality score and more false-positive base calls) as compared with a less stringent cut-off in 5 out of 11 samples where schmutzi’s filtering was effective. In fact, we strongly recommend applying a series of quality filters with increasing stringency for each sample and determining the most suitable cut-off for each sample, which preserves as many true-positive base calls (indicated by a high-HaploGrep-quality score) (Figure 1 and Figure S1, Table S2) while filtering out as many false-positive base calls as possible (as indicated by the number of “global private mutations” noted in HaploGrep’s report) (Figure 3 and Figure S2, Table S2).

Similarly, ANGSD’s “*-trim*” option achieves almost an equally good (in most cases) filtering of possibly erroneous bases at read termini by trimming *n* number of bases at both ends. As in the case of schmutzi’s base quality score, there is no ideal value for the number of bases trimmed, as this depends on the specific sample. We highly recommend the use of this filter when analyzing aDNA sequence data, applying the filter increasingly (i.e., trimming of 1, 3, 5, etc., terminal bases) until the highest haplogroup prediction score is achieved. Interestingly, ANGSD’s base-quality filter (*-minQ*) did not have any substantial improvement in the consensus sequence quality over and above the aforementioned trimming filter for most samples (Figure 1 and Figure S1, Table S2).

Overall, where schmutzi was able to run, it tended to create consensus sequences of slightly better quality than ANGSD for most samples (Figure 2), while in some cases (e.g., sample ZMOJ) the difference between the two tools was substantial. For lower-quality samples though (e.g., KFH2, KRD003), and given that both tools could run properly, ANGSD tended to give better quality sequences (Figure 2).

The main detrimental factors influencing the success of quality filtering for both tools, but particularly for schmutzi, appear to be the low number of available sequence reads and bases and, to a smaller extent, high damage/deamination at 5′ end position (Figure 4 and Figure S3, Table S3). It is advisable that for such low-quality samples, different tools designed for calling the mtDNA consensus sequence from raw genomic data are used, and the sequence showing the best quality (best overall haplogroup quality score and lower number of global private mutations reported by HaploGrep) is used. A point of caution here is that even in the presence of a seemingly valid mitogenome consensus sequence, it is advisable not to rely at all on samples with mean coverage < 0.30X and a total number of reads of <1.5 million. This is based on the characteristics of the very low-coverage sample KRD003 analyzed here, from which we could marginally derive a valid consensus sequence, with anything lower than that providing problematic sequences and haplogroup predictions. Generally, it is advisable that the predicted haplogroup needs to be checked carefully in context of archeological space and time. For example, some of the newly derived mtDNA haplogroups in our analysis do not seem to provide any useful information, and the possibility of a false haplogroup call is very likely (further details in the following subsection).

### 3.3. Newly Predicted mtDNA Haplogroups and Subclades 

In the process of quality testing the mitochondrial consensus sequences derived in the current analysis, we have slightly improved the haplogroup resolution of some samples, namely sample ZBC classified from K2b to K2b2 (HaploGrep highest overall quality achieved 0.945), sample ZMOJ classified from K1a to K1a19 (highest overall quality achieved 0.996), samples ZKO, ZHJ, and ZHAJ from U3 to U3b (highest overall quality achieved 0.962, 0.938, and 0.947, respectively), sample KFH2 from N1a1b to I6 (highest overall quality achieved 0.801), and sample MTT001 from U7 to U7b (highest overall quality achieved 0.970). A possible reason for not reporting these subclades in the original publications is probably that our newly derived haplotypes, following a more systematic quality-filtering approach and two alternative tools, resulted in more predictive haplotypes than originally achieved (only applicable where the highest-archived HaploGrep quality score exceeds the one reported in the original study). Another reason may be the way HaploGrep was used to predict mtDNA haplogroups. More specifically, even though in a Linux environment, HaploGrep is quite fast and efficient in handing multiple samples at once, it only provides the single most highly ranked predicted haplogroup per sample. The issue with this approach is that the second most highly ranked haplogroup might be a slightly deeper subclade of the highest-ranked prediction (e.g., highest-ranked prediction K2b, second-highest-ranked prediction K2b2). In some cases, this second-highest-ranked deeper subclade may have an identical quality score or one very slightly lower than the original perdition, i.e., the possibility of the deeper subclade (e.g., K2b2) is as likely as the more basal prediction (e.g., K2b). If HaploGrep is not used in its web application as well, such deeper subclade classification might be missed.

Additionally, we provide newly predicted haplogroups for six previously unassigned samples from the Early/Middle Chalcolithic site of Tell Kurdu in Southern Anatolia/Northern Levant. More specifically, sample KRD001 was predicted to belong to mtDNA haplogroup P5, sample KRD002 to haplogroup N1a1a, sample KRD003 to haplogroup U3b, sample KRD004 to haplogroup F1a1a1*2, sample KRD005 to haplogroup U8b, and sample KRD006 to haplogroup H20a.

With the exception of sample KRD003, which had the highest coverage (still very low at 0.35×) from all the aforementioned samples and thus provided the highest HaploGrep quality score (0.913), all other newly predicted haplogroups should be interpreted with great caution. In particular, the predicted haplogroups P5 (sample KRD001) and F1a1a1*2 (sample KRD004) seem too basal, as well as inconsistent with the archeological and geographical context of these samples and are thus highly unlikely. In addition, although the other predicted haplogroups, namely N1a1a (sample KRD002), U8b (sample KRD005), and H20a (sample KRD006), seem more realistic as regards the given archeological context, they appear to miss several key haplogroup-defining polymorphisms.

Regarding sample KRD003 (Early Chalcolithic Tell Kurdu, Southern Anatolia/Northern Levant), the only newly predicted haplogroup we report with relatively high confidence, mtDNA haplogroup U3b seems highly realistic, as it appears in three other Anatolian Neolithic individuals analyzed in the current study (ZKO, ZHJ, and ZHAJ) plus one previously reported, all from the Anatolian Aceramic Neolithic site of Boncuklu in Southern Anatolia. Additionally, this haplogroup has also been found in other Anatolian Neolithic sites, such as Çatalhöyük [36] and Barcin [5]. Interestingly, mtDNA haplogroup U3 acts as a good indicator of the spread of agriculture from Anatolia to Europe, as it has also been identified in early Neolithic European farmers [5,37,38].

### 3.4. Implications for Downstream Ancient mtDNA Phylogenetic Analysis 

The ultimate aim of our analysis was to determine a valid and straight-forward approach for deriving ancient mitogenomes from raw genomic data to be used in subsequent downstream phylogenetic analysis, post-reconstruction. The implications of our findings should be considered in context of what researchers aim to achieve from a reconstructed ancient mitogenome sequence. Overall, the performance of both tested tools is compromised in samples with a low available number of sequence reads, to the extent of either not running or providing erroneous and inaccurate consensus sequences. In such cases, any downstream mtDNA analyses should be performed with extreme caution and, for very low-quality samples, should be avoided altogether.

In cases where researchers are just interested in deriving the mtDNA haplogroup of ancient samples, this can be performed with high accuracy in samples with >1,000,000 total sequence reads available, based on our findings. If, however, there is interest in deeper downstream mtDNA phylogenetic and phylogeographic analyses, such as intrapopulation (e.g., different standard population genetic indices, as well as kinship analysis) and interpopulation (e.g., shared haplotypes between populations, as well as analysis of molecular variance for estimation of population subdivision and genetic distances between populations, i.e., pairwise FST indices), more stringent sample quality criteria should be used to avoid erroneous findings and misleading conclusions. Based on our findings, it seems that aDNA samples with >3,000,000 total reads and >200,000,000 total bases available (or >1000 mitochondrial reads and >50,000 mitochondrial bases) and, at the same time, not suffering from extensive deamination or exogenous contamination, could potentially provide very high quality ancient mitochondrial haplotypes. These could be safely used for such downstream analyses, always considering the fact that possible minor errors or inaccuracies are more likely in aDNA analysis compared with modern DNA analysis.

The wealth of publicly available ancient genome data is an invaluable resource for studying, in depth, ancient human migrations and elucidating unknown and highly debated aspects of human history and evolution [33,39,40]. Over the past few years, whole-genome data received the ‘lion’s share’ in aDNA findings, something reflected in the scarcity of publicly available complete mitogenomes in the relevant datasets. This gap in publicly available ancient mitogenome data compromises the holistic and in-depth analysis of aDNA, combining both autosomal and uniparental markers. Although the vast majority of high-impact aDNA publications [5,6,41,42,43,44] do provide uniparental information about the analyzed ancient individuals, in the case of mtDNA this is usually limited to reporting of the basic haplogroup, with very few studies reporting findings from more detailed sequence-based mitochondrial analysis and haplogroup assignment scores [40,45].

It should be noted that major milestones in the field of archaeogenetics, with substantial contribution in elucidating important aspects of human migration and evolution, come from phylogenetic analysis of ancient mtDNA. Some examples are the revolutionary discovery that early European Neolithic farmers were matrilineally distinct from their contemporary hunter–gatherers and derived primarily from the same maternal gene pool as Near Eastern Neolithic farmers [11,12]. Additionally, ancient mitogenomes revealed for the first time the massive migration of steppe pastoralist into Europe during the Early Bronze Age [37,46] and, more recently, a parallel migration from similar groups at approximately the same time in Central/Eastern Asia [47].

An additional advantage of having available rich mtDNA data for downstream analysis in ancient samples is the unique ability to investigate kinship and identify close relationships between sampled individuals. For example, ancient mitogenomes revealed the absence of maternal kinship in close burials at the site of Çatalhöyük in Neolithic Anatolia [36], while a recent revolutionary study revealed, using a combination of autosomal and uniparental aDNA, complex family dynamics and social bonds in Neolithic Britain, characterized by strong virilocal burial practices and female exogamy, with kinship likely to encompass social bonds independent of biological relatedness [17].

## 4. Materials and Methods

### 4.1. Choice of Samples and Retrieval of Raw Genomic Data 

The criteria for the choice of ancient samples for the purposes of addressing the aims of the current study were: (a) the ancient samples were relatively old (Late Bronze Age and earlier); (b) raw mtDNA haplotypes along with information on mtDNA haplogroup were publicly available; (c) sample haplogroup prediction quality scores were also publicly available; and (d) library preparation method incorporated a Uracyl DNA glycosylase–endonuclease treatment to partially remove deaminated Cytosine residues in DNA strands while still retaining them in terminal positions.

Following a thorough search of the literature, we identified two publications which fulfilled these criteria [29,30]. The first included 8 ancient genomes, comprising a Late Pleistocene (Epipaleolithic) Anatolian hunter–gatherer (ZBC) from the site of Pinarbași directly dated to 13,642–13,073 cal BCE, 5 early Neolithic Aceramic Anatolian farmers (ZHAG, ZMOJ, ZKO, ZHJ, and ZHAJ) from the site of Boncuklu dated to 8300–7800 BCE, and 2 Early Neolithic (PPNB) southern Levantine farmers from the sites of Kfar HaHoresh (KFH2) and Ba’ja (BAJ001), directly dated to c. 7700–7600 cal BCE and c. 7027–6685 cal BCE, respectively [29] (Table 1 and Table S1). An additional benefit of choosing these specific samples was that all mtDNA haplotypes were manually revised using Integrative Genomics Viewer (IGV) and unreliable positions, representing likely incorrect base calls, were discarded (for more detail see supplementary info in [29]).

The second identified publication included 110 ancient samples ranging from the Late Neolithic to the Bronze Age from Anatolia, Levant, and the Caucasus. From these samples, we chose the 9 oldest samples. Although additional samples could be derived from this publication, the in-depth analysis conducted here is more suitable for a relatively low number of samples. These 9 ancient genomes comprised an Early Chalcolithic individual (CBT018) from the site of Boğazköy-Büyükkaya, Central/Northern Anatolia, dated to 7576–7463 cal BCE, 2 Caucasus lowlands Late Neolithic individuals (MTT001, POT002) from the sites of Mentesh Tepe (dated to 7679–7594 cal BCE) and Polutepe (dated to 7458–7323 cal BCE), respectively, and 6 Southern Anatolia/Northern Levant Early/Middle Chalcolithic individuals (KRD001, KRD002, KRD003, KRD004, KRD005, and KRD006) from the site of Tell Kurdu, dated to 7750–6799 cal BCE (Table 1).

Raw genomic data from the aforementioned 17 ancient samples were retrieved from the European Nucleotide Archive (ENA) at EMBL-EBI using the following accession links: https://www.ebi.ac.uk/ena/browser/view/PRJEB24794 (accessed on 6 October 2021) and https://www.ebi.ac.uk/ena/browser/view/PRJEB37213 (accessed on 9 February 2022).

For applying the schmutzi pipeline, we retrieved data in FASTQ format, while for the ANGSD pipeline, we retrieved the same data in BAM format, based on the instructions of each tool [24,28]. For the 8 samples derived from the first publication [29], only the whole-genome raw data (1240K capture) were publicly available, which include the MT chromosome, while for the remaining 9 samples [30] direct mitochondrial genomic data (mitocapture) were publicly available. The implication of this was that an additional step was required (isolating the MT chromosome from whole-genome data) for 8 out of 17 samples (discussed further in the relevant subsection below).

### 4.2. Reconstructing Ancient Mitogenome Sequences from Raw Genomic Data 

#### 4.2.1. Schmutzi Pipeline

The schmutzi tool (version 1.5.6 used here) and pipeline has been explicitly designed to detect exogenous human contamination in ancient mtDNA. While using sequence deamination patterns and fragment length distributions, it reconstructs the endogenous mitogenome consensus sequence in the process, even when contamination reaches 50% and above. This procedure is split into two parts, one performing a damage-based (deamination patterns) contamination estimation (contDeam application) and a second one (schmutzi application) utilizing a database of 197 mitochondrial allele frequencies or a subset of Eurasian allele frequencies, which actually call the mitogenome consensus sequence for both the endogenous and contaminant human DNA present in a given sample [24].

The schmutzi pipeline followed to call the mitogenome consensus sequence from raw genomic data can be found in Figure S4, and the relevant code is available in Appendix A. In order to prepare the raw genome samples for analysis, we aligned the FASTQ raw sequence data to the reference mitochondrial sequence (revised Cambridge Reference Sequence-rCRS) using BWA *aln* (RRID:SCR_010910, v0.7.17) [48]. It should be noted here that in cases where the mitogenome is not available as a separate FASTQ file and whole genome data are used, the alignment should be performed only on the mitochondrial reference sequence, not the whole reference genome. BWA *samse* was used to create a SAM file from the output of the previous step (works only for single-end reads). Following this, sequencing reads were realigned by CircularMapper [49], which performs an improved mapping to circular reference genomes, such as mtDNA, and provides an aligned BAM file. Although this step is not essential in the original pipeline, it is anticipated to provide higher-quality consensus sequences, given that many phylogenetically informative positions can be found at the beginning and the end of the mtDNA reference sequence [49]. SAMtools’ (RRID:SCR_002105, v1.15) *sort* was used for sorting the aligned BAM file. An additional step that aids schmutzi to access the data in the BAM file is MD tagging. MD tags are commonly used to aid accessing to specific loci/regions of the genome, without the need for a reference genome, given that the tag contains information on positions in the relevant read with matches, mismatches or indels. MD tags were added to the aligned and sorted BAM file using SAMtools’ *calmd*. The MD-tagged BAM file was then indexed using SAMtools’ *index*.

Following the above steps and according to the developer’s recommended workflow for ancient samples, we first called the contDeam application to estimate initial contamination and endogenous deamination rates. Following this, we called the actual schmutzi application to run the iterative procedure without the prediction of the contaminant (*--notusepred*) and repeated the process with the prediction of the contaminant. Following the developer’s recommendations, in cases where the contamination estimate was more than a few percent, we rerun the procedure using the “*--uselength*” option (the program is set to use the length of the molecules).

The output of the above process contains the final estimate of exogenous human contamination and the consensus mitogenome sequences for both the endogenous and the contaminant DNA, as well as a log file with the raw output produced while inferring the endogenous consensus sequence, including the per-base likelihood for each position of being the endogenous base (e.g., likelihood of true positive base call), as estimated by a Bayesian approach. It should be noted that, although deamination is taken into consideration while calling these initial sequences, no base quality filtering is applied, and these should therefore not be used for any downstream analysis.

#### 4.2.2. ANGSD Pipeline

Unlike schmutzi, ANGSD (version 0.936 used here) is a multipurpose analysis tool for next generation sequencing data [28], which has not been explicitly designed for handling ancient genomic data. ANGSD does incorporate, however, a very handy and straight-forward option *(-dofasta*), which calls the consensus sequence from raw sequencing data (BAM file). The function uses genome information in the BAM header to determine the length and chromosome names. As in the case of schmutzi, for sites where no information is available an “N” is incorporated in the sequence. ANGSD has 3 alternative options in calling the consensus sequence: option 1, where a random base is sampled from the reads at each position; option 2, where the most common base is selected (in case of ties, a random base is chosen among the bases with the same maximum counts); and option 3, where the base with the highest effective depth (the product of the mapping quality and scores) is selected. We have tested all three options in a selection of samples, and it was apparent that the called consensus sequence derived from option 1 was inferior to those derived from options 2 and 3 (data not shown). We eventually opted for option 2, as it allows for more filtering options (refer to subsection below). It should be noted that this option requires an additional option for allele counts (*-doCounts*) in order to determine the most common base in the specific site.

The ANGSD pipeline followed to call the mitogenome consensus sequence from raw genomic data can be found in Figure S4, and the relevant code is available in Appendix A. In order to proceed with calling the mitogenome consensus sequence, we first had to isolate the mitochondrial (MT) chromosome from the whole-genome BAM file (wherever the mitogenome was not available as a separate BAM file). In order to perform this, after first checking that indeed the BAM file was sorted and creating a BAM index file (BAI), using SAMtools (*stats* and *index*, respectively), we isolated the MT chromosome from the whole genome using SAMtools’ *view*. Following this, ANGSD was used in a simple step specifying the output (*-out*) and input (*-i*) files, followed by options *-doFasta 2* and *-doCounts 1*. This step creates a fasta.gz file, which when extracted provides the complete consensus sequence in FASTA format. As in the case of schmutzi, this initial-derived consensus sequence has not been quality filtered, therefore it is anticipated to be of lower quality given the common pitfalls of ancient DNA analysis, namely deamination and contamination.

### 4.3. Filtered Consensus Calling for Quality Improvement of Reconstructed Mitogenomes

Despite the wealth of availability of ancient human samples, cytosine deamination and exogenous contaminant DNA characterizing aDNA complicates the accurate identification of endogenous mtDNA for downstream analysis. Both tools of choice, in the current study, have quality filters incorporated for improving the accuracy of consensus sequence calling and dealing with ancient DNA damage/deamination (schmutzi and ANGSD) and contamination (schmutzi).

#### 4.3.1. Schmutzi Filtering

Schmutzi’s “log2fasta” application can be used on the final endogenous sequence log file (refer to previous subsection) to obtain a consensus sequence at various quality cutoffs based on the quality score (q score) of each base call. The q score provides the probability that a base is called incorrectly (i.e., false positive call), as estimated by a phredlike algorithm [50,51]. The q score is defined as: Q = −10 log_10_(e), where “e” is the estimated false-positive base call probability. As expected, lower Q-scores indicate higher probability of a false-positive base call, rendering bases unusable and increasing the likelihood of biased findings in downstream analysis.

In order to investigate the effectiveness of these base-quality filters using “log2fasta” from the schmutzi package, we used cut-offs with increasing stringency, (*-q 5*, *-q 10*, *-q 15*, *-q 20*, *-q 25*, *-q 30*, *-q 40*, and *-q 50*) and checked whether we still had a sufficient number of base calls. It was apparent that post Q30, the number of bases in the sequence decreased dramatically for the majority of samples in our study, thus, we chose Q30 as the most stringent quality cut-off. It should be noted that a Q-score of 30 denotes a probability for a false-positive base call of 1 in 1000, thus corresponding to a base-call accuracy of 99.9%. Similarly, Q-scores of 20 and 10 correspond to base-call accuracies of 99% and 90%, respectively.

An interesting point about applying increasing stringency in base-quality filtering is that more stringent cut-offs (e.g., Q30) are anticipated to have a dual effect, i.e., on one hand they tend to correctly filter out false-positive base calls, but on the other hand they may also filter out true-positive calls and thus compromise the accuracy of the derived consensus sequence. Thus, our approach attempts to determine, for each sample, an acceptable threshold between filtering erroneous base calls and reserving enough valid information in the consensus sequence.

#### 4.3.2. ANGSD Filtering

As in the case of schmutzi, ANGSD has the capability of filtering calls based on base-quality scores. In this case, this is achieved by the “*-minQ”* option, which sets the minimum base quality (e.g., 10), discarding bases with quality scores below that threshold. It should be noted that the default base-quality cut-off of ANGSD’s “*-dofasta*” is 13. For this reason, and since we wanted to determine the effect of increasing levels of base quality filtering (5, 10, 15, 20, 25, and 30), we set the “*-minQ*” option to 0 for deriving the totally unfiltered consensus sequence.

Since ANGSD does not incorporate a default pipeline for taking deamination into consideration as schmutzi does, it requires an option to deal with the increasing fragmentation and damage observed in aDNA samples. This comes in the form of the “*-trim*” option, which utilizes the fact that nucleotide misincorporations due to damage in aDNA tend to cluster at read termini [52], systematically trimming a set number of bases at read ends. This is anticipated to improve the quality of the derived consensus mitogenome sequence. Due to the fundamental need for this type of filtering in aDNA, we have first applied the trimming filter in all samples with increasing stringency (1, 3, and 5 terminal bases trimmed at both read ends) in order to determine the option which provides the best balance between filtering out false-positive base calls and reserving as many true-positive calls as possible. Once this was determined for each sample, the base-quality filter was applied as described above.

### 4.4. Determination of mtDNA Haplogroup Prediction Accuracy and Sample Quality 

The reconstructed mitogenome sequences for all samples (both quality filtered at different levels and unfiltered), derived by both tools following the above procedures, were used to predict mtDNA haplogroups using HaploGrep 2 (version 2.4.0) [31] (*haplogrep classify*).

HaploGrep performs haplogroup classification from an input haplotype or complete sequence, based on precalculated phylogenetic weights that correspond to the occurrence per position in Phylotree and reflect the mutational stability of a variant. Haplogrep 2 improves on the original algorithm by incorporating a generic rule-based system for immediate quality control of the input sample. This allows detecting artificial recombinants and missing variants, as well as annotating rare and phantom mutations [31].

HaploGrep’s overall quality score is calculated based on the Kulczynski measure, defined as follows:

(Haplogroup Weight + Sample Weight) × 0.5,(1)

This formula can be broken down to:

Haplogroup Weight = Found Polymorphisms Weight/Expected Polymorphisms Weight (2)

and:

Sample Weight = Found Polymorphisms Weight/Sample Polymorphisms Weight(3)

In the above, “Found Polymorphisms” refers to polymorphisms from the input haplotype that are characteristic in the predicted haplogroup, “Expected Polymorphisms” refers to the complete set of polymorphisms that are expected for the predicted haplogroup, and “Sample Polymorphisms” refers to polymorphisms that are included in the sample and fall into the specified range. Higher HaploGrep quality scores indicate higher quality of the consensus sequence (fewer erroneous base calls). As expected, the higher the number of true positives base calls, the higher the likelihood of correctly identified polymorphisms for a given haplogroup (higher “Found Polymorphisms” weight). On the contrary, a derived mitogenome sequence containing a high number of false-positive base calls results in low “Found Polymorphisms” weight relative to “Expected Polymorphisms” weight and an overall low-quality score. Overall, HaploGrep’s quality score ranges from 0.5 to 1, where 0.5 indicates no expected polymorphisms found and 1 is a perfect match (all expected polymorphisms for the predicted haplogroup found and no possible erroneous base calls detected).

In our case, HaploGrep’s overall quality score served the purpose of validating the accuracy of mitogenome reconstruction from raw read data by the two tools of choice, with the overall quality for each sample, as reported in the original study, being treated as the reference method. It should be noted that in 8 out of 17 samples (ZBC, ZHAG, ZMOJ, ZKO, ZHJ, ZHAJ, KFH2, and BAJ001) initially derived haplotypes were manually revised using IGV in the analysis of the original study [29], thus they can be safely considered as the gold standard for comparing the validity of our newly derived filtered haplotypes. Additionally, it should be noted that for 6 out of 17 samples (KRD001, KRD002, KRD003, KRD004, KRD005, and KRD006) no quality score was available to use as reference, as it was not provided in the original study [30] due to the very low coverage of these samples.

Additional to the overall quality score, HaploGrep reports two other very useful pieces of information, namely the number of “missed expected polymorphisms” and the number of “global private mutations not known by Phylotree”. The first represents the total number of expected polymorphisms in the predicted haplogroup, which were not detected in the given haplotype (the higher the number of missed expected polymorphisms, the lower the quality of the consensus sequence). The second represents the total number of polymorphisms detected in the given haplotype, which are unknown in the current phylogeny, very likely indicating an incorrect base call during the process of consensus sequence calling. The higher the number of probable incorrect base calls, the lower the quality of the consensus sequence.

## 5. Conclusions

Our study provides, for the first time, a systematic overview of the performance of two widely used consensus sequence calling tools, clearly indicating the value of systematically applied quality filtering in reconstructing complete ancient mitogenomes, given the availability of sufficient raw sequence data and low levels of sample deamination and exogenous contamination. The schmutzi pipeline, taking into consideration endogenous deamination and exogenous contamination, appeared to have the edge over the alternative method (ANGSD) for reconstructing accurate aDNA mitogenome consensus sequences in moderate- to high-coverage samples (>1,000,000 reads). ANGSD, however, while being much faster and user-friendly, has the ability to reconstruct acceptable quality aDNA consensus sequences through its read termini trimming filter, even in low-coverage samples. Both tools, but particularly schmutzi, are limited by the very low availability of sequence reads and bases, highlighting a point of caution for performing downstream mtDNA analyses on low-quality aDNA samples. Overall, our findings provide a valuable resource and guidance for researchers aiming to utilize new or previously published raw genomic data for conducting deeper phylogenetic, phylogeographic, and kinship analyses based on ancient human mitochondrial DNA.

## Figures and Tables

**Table 1:** Overview of ancient samples analyzed in the current study with details on coverage, damage, and contamination, including updated haplogroup predictions.

Source	Sample	Site	Location and Era	Sex	Estimated Date	Total Read Count	Total Base Count	Endogenous DNA (%)	Mean Coverage (Fold)	Damage (5′ Terminal)	Mt Contamination	Μt Haplogroup (Original Study)	Mt Haplogroup (Current Study)
[29]	ZBC	Pinarbași	Anatolia Epipaleolithic	M	13642–13073 cal BCE	10,036,367	638,989,878	2.42	2.90	0.09	0.01 (0.00–0.02)	K2b	K2b2
[29]	ZHAG	Boncuklu	Anatolia Aceramic Neolithic	F	8300–7800 BCE	8,127,772	488,875,951	2.14	1.48	0.11	0.01 (0.00–0.02)	N1a1a1	N1a1a1
[29]	ZMOJ	Boncuklu	Anatolia Aceramic Neolithic	M	8300–7800 BCE	3,513,636	176,572,167	1.03	0.80	0.22	0.03 (0.02–0.04)	K1a	K1a19
[29]	ZKO	Boncuklu	Anatolia Aceramic Neolithic	M	8300–7800 BCE	4,594,498	257,455,118	1.51	0.90	0.16	0.01 (0.00–0.02)	U3	U3b
[29]	ZHJ	Boncuklu	Anatolia Aceramic Neolithic	F	8300–7800 BCE	5,063,868	290,873,189	2.36	0.76	0.09	0.01 (0.00–0.02)	U3	U3b
[29]	ZHAJ	Boncuklu	Anatolia Aceramic Neolithic	F	8269–8210 cal BCE	3,848,792	216,286,212	1.48	0.69	0.16	0.03 (0.02–0.04)	U3	U3b
[29]	KFH2	Kfar HaHoresh	Levant Early Neolithic (PPNB)	F	7712–7589 cal BCE	1,315,907	58,245,650	0.20	0.16	0.26	0.06 (0.04–0.08)	N1a1b	I6
[29]	BAJ001	Ba’ja	Levant Early Neolithic (PPNB)	F	7027–6685 cal BCE	2,038,720	104,654,562	5.51	0.75	0.28	0.01 (0.00–0.02)	N1b1a	N1b1a
[30]	CBT018	Boğazköy-Büyükkaya	C./N. Anatolia ^1^ Early Chalcolithic	F	7576–7463 cal BCE	3,814,623	222,533,552	5.19	0.51	0.14	0.01 (0.00–0.03)	X2	X2
[30]	MTT001	Mentesh Tepe	Caucasus lowlands Late Neolithic	F	7679–7594 cal BCE	11,887,457	582,094,002	14.08	0.55	0.21	0.03 (0.01–0.05)	U7	U7b
[30]	POT002	Polutepe	Caucasus lowlands Late Neolithic	F	7458–7323 cal BCE	8,339,513	392,934,499	6.63	0.41	0.31	0.02 (0.00–0.04)	H13a2b	H13a2b
[30]	KRD001	Tell Kurdu	S. Anatolia/N. Levant ^2^ Early Chalcolithic	M	7670–7590 cal BCE	678,068	37,097,407	0.19	0.20	0.22	0.01 (0.00–0.03)	n/a ^3^	*P5*
[30]	KRD002	Tell Kurdu	S. Anatolia/N. Levant ^2^ Middle Chalcolithic	M	6955–6799 cal BCE	520,046	25,733,234	0.26	0.15	0.27	0.01 (0.00–0.03)	n/a ^3^	*N1a1a*
[30]	KRD003	Tell Kurdu	S. Anatolia/N. Levant ^2^ Early Chalcolithic	M	7656–7572 cal BCE	1,646,820	83,050,541	0.74	0.35	0.26	0.03 (0.00–0.11)	n/a ^3^	U3b
[30]	KRD004	Tell Kurdu	S. Anatolia/N. Levant ^2^ Levant Early Chalcolithic	F	7664–7582 cal BCE	236,781	12,662,467	0.22	0.06	0.21	0.02 (0.00–0.06)	n/a ^3^	F1a1a1*2
[30]	KRD005	Tell Kurdu	S. Anatolia/N. Levant ^2^ Early Chalcolithic	M	7706–7614 cal BCE	132,930	7,367,352	0.13	0.03	0.18	0.01 (0.00–0.03)	n/a ^3^	*U8b*
[30]	KRD006	Tell Kurdu	S. Anatolia/N. Levant ^2^ Early Chalcolithic	F	7750–7350 BCE	1,060,964	60,413,143	1.15	0.21	0.19	0.03 (0.01–0.05)	n/a ^3^	*H20a*

^1^ C./N. Anatolia (Central/Northern Anatolia). ^2^ S. Anatolia/N. Levant (Southern Anatolia/Northern Levant). ^3^ A mitochondrial haplogroup was not reported in the original publication; mitochondrial haplogroups shown in *italics* should be interpreted with caution.

## Data Availability

The raw genomic data (FASTQ/BAM files) used for the current analysis are publicly available at the European Nucleotide Archive using the following links: https://www.ebi.ac.uk/ena/browser/view/PRJEB24794 (accessed on 6 October 2021) and https://www.ebi.ac.uk/ena/browser/view/PRJEB37213 (accessed on 9 February 2022).

## Supplementary Materials

The following supporting information can be downloaded at: https://www.mdpi.com/article/10.3390/ijms23094651/s1.

## Appendix A. Codes (Command Lines) for Reconstructing Mitogenome Sequences from Raw Genomic Data Using Two Alternative Tools (Schmutzi and ANGSD)

*Schmutzi Pipeline*
##STEP 1: Sample preparation using BWA and SAMtools##
#1a: Align FASTQ sample to reference fasta and create a SAM file
bwa aln MT_reference_file input_file.fastq > input_file.sai
bwa samse MT_reference_file input_file.sai input_file.fastq > output_file_MTaligned.sam
#1b (optional): Realignment to mtDNA reference by CircularMapper (automatic conversion to BAM)
java -jar realign-1.93.5.jar -e 500 -i output_file_MTaligned.sam -r MT_reference_file.fa
#1c: Sort BAM file (use aligned BAM file derived in previous step)
samtools sort output_file_MTaligned _CM_realigned.bam -o output_file_MTaligned _CM_realigned_sort.bam
#1d: MD-Tagging (use aligned and sorted BAM file derived in previous step)
samtools calmd -b output_file_MTaligned _CM_realigned_sort.bam MT_reference_file.fa > output_file_MTaligned _CM_realigned_sort_MD.bam
#1e: Index the MD-tagged BAM file
samtools index output_file_MTaligned _CM_realigned_sort_MD.bam
##STEP 2: Estimate endogenous deamination using contDeam##
contDeam.pl --library double --out output_name output_file_MTaligned _CM_realigned_sort_MD.bam
##STEP 3: Estimate contamination and call the (unfiltered) consensus sequence using schmutzi##
#3a: First run the iterative procedure without the prediction of the contaminant
schmutzi.pl --notusepredC --uselength --ref MT_reference_file.fa --out output_name_npred freqs output_name output_file_MTaligned _CM_realigned_sort_MD.bam
#3b: Then repeat the above with the prediction of the contaminant
schmutzi.pl --uselength --ref MT_reference_file.fa --out output_name_npred freqs output_name output_file_MTaligned _CM_realigned_sort_MD.bam
##STEP 4: Apply filtering in the consensus sequence derived in the previous step using log2fasta##
#Use the log file created via running the schmutzi application to filter by base Q-score (in this case discards bases with Q-score below 10-repeat with different cut-offs)
log2fasta -q 10 output_name_npred_final_endo.log > output_name_filtered_schmu_q10.fasta

*ANGSD Pipeline*
##STEP 1: Sample preparation using SAMtools##
#1a: check whether the input BAM file is sorted
samtools stats input_file.bam | grep “is sorted:”
#1b: create a BAM index file (BAI)
samtools index input_file.bam input_file.bai
#1c: Isolate the MT chromosome as a separate BAM file (this step will not be needed if the mitocapture BAM file is available)
samtools view -b input_file.bam MT > input_file_MT.bam
##STEP 2: Call the consensus sequence using ANGSD (no filtering)##
#2a: Call the mitogenome consensus sequence from the MT BAM file (option 1: use the most common base)
angsd -out output_file -i input_file.bam -doFasta 2 -doCounts 1
#2b (optional): Call the mitogenome consensus sequence from the MT BAM file (option 2: use the base with the highest effective depth-EBD)
angsd -out output_file -i input_file.bam -doFasta 3
##STEP 3: Call the consensus sequence using ANGSD (with filtering)##
#3a: Call the mitogenome consensus sequence from the MT BAM file (option 1) and filter by trimming the first/last 1 base of each read (in this case trims first/last base of each read-repeat by setting different n of bases to be trimmed)
angsd -out output_file -i input_file.bam -doFasta 2 -doCounts 1 -trim 1
#3b: Call the mitogenome consensus sequence from the MT BAM file (option 1) and filter by trimming the first/last 1 base of each read *and* setting minimum base quality to 10 (in this case discards base quality below 10-repeat with different cut-offs)
angsd -out output_file -i input_file.bam -doFasta 2 -doCounts 1 -trim 1 -minQ 10

*HaploGrep*
#mtDNA Haplogroup prediction using Haplogrep 2, based on the fasta sequence files derived in the above pipelines
haplogrep classify --extend-report --format fasta --in output_file.fasta --out output_file.txt

## References

[1] Handt O., Richards M., Trommsdorff M., Kilger C., Simanainen J., Georgiev O., Bauer K., Stone A., Hedges R., Schaffner W. (1994). Molecular genetic analyses of the Tyrolean Ice Man. Science.

[2] Vernesi C., Caramelli D., Dupanloup I., Bertorelle G., Lari M., Cappellini E., Moggi-Cecchi J., Chiarelli B., Castrì L., Casoli A. (2004). The Etruscans: A population-genetic study. Am. J. Hum. Genet..

[3] Sampietro M.L., Caramelli D., Lao O., Calafell F., Comas D., Lari M., Agusti B., Bertranpetit J., Lalueza-Fox C. (2005). The genetics of the pre-Roman Iberian Peninsula: A mtDNA study of ancient Iberians. Ann. Hum. Genet..

[4] Haak W., Forster P., Bramanti B., Matsumura S., Brandt G., Tanzer M., Villems R., Renfrew C., Gronenborn D., Alt K.W. (2005). Ancient DNA from the first European farmers in 7500-year-old Neolithic sites. Science.

[5] Mathieson I., Lazaridis I., Rohland N., Mallick S., Patterson N., Roodenberg S.A., Harney E., Stewardson K., Fernandes D., Novak M. (2015). Genome-wide patterns of selection in 230 ancient Eurasians. Nature.

[6] Patterson N., Isakov M., Booth T., Büster L., Fischer C., Olalde I., Ringbauer H., Akbari A., Cheronet O., Bleasdale M. (2022). Large-scale migration into Britain during the Middle to Late Bronze Age. Nature.

[7] Liu Y., Mao X., Krause J., Fu Q. (2021). Insights into human history from the first decade of ancient human genomics. Science.

[8] Orlando L., Allaby R., Skoglund P., Der Sarkissian C., Stockhammer P.W., Ávila-Arcos M.C., Fu Q., Krause J., Willerslev E., Stone A.C. (2021). Ancient DNA analysis. Nat. Rev. Methods Primers.

[9] Kivisild T. (2015). Maternal ancestry and population history from whole mitochondrial genomes. Investig. Genet..

[10] Fu Q., Mittnik A., Johnson P.L., Bos K., Lari M., Bollongino R., Sun C., Giemsch L., Schmitz R., Burger J. (2013). A revised timescale for human evolution based on ancient mitochondrial genomes. Curr. Biol..

[11] Bramanti B., Thomas M.G., Haak W., Unterländer M., Jores P., Tambets K., Antanaitis-Jacobs I., Haidle M.N., Jankauskas R., Kind C. (2009). Genetic discontinuity between local hunter-gatherers and central Europe’s first farmers. Science.

[12] Haak W., Balanovsky O., Sanchez J.J., Koshel S., Zaporozhchenko V., Adler C.J., Der Sarkissian C.S., Brandt G., Schwarz C., Nicklisch N. (2010). Ancient DNA from European early neolithic farmers reveals their near eastern affinities. PLoS Biol..

[13] Fernández E., Pérez-Pérez A., Gamba C., Prats E., Cuesta P., Anfruns J., Molist M., Arroyo-Pardo E., Turbón D. (2014). Ancient DNA analysis of 8000 BC near eastern farmers supports an early neolithic pioneer maritime colonization of Mainland Europe through Cyprus and the Aegean Islands. PLoS Genet..

[14] Posth C., Renaud G., Mittnik A., Drucker D.G., Rougier H., Cupillard C., Valentin F., Thevenet C., Furtwängler A., Wißing C. (2016). Pleistocene mitochondrial genomes suggest a single major dispersal of non-Africans and a Late Glacial population turnover in Europe. Curr. Biol..

[15] Pala M., Olivieri A., Achilli A., Accetturo M., Metspalu E., Reidla M., Tamm E., Karmin M., Reisberg T., Kashani B.H. (2012). Mitochondrial DNA signals of late glacial recolonization of Europe from near eastern refugia. Am. J. Hum. Genet..

[16] Vai S., Amorim C.E.G., Lari M., Caramelli D. (2020). Kinship determination in archeological contexts through DNA analysis. Front. Ecol. Evol..

[17] Fowler C., Olalde I., Cummings V., Armit I., Büster L., Cuthbert S., Rohland N., Cheronet O., Pinhasi R., Reich D. (2022). A high-resolution picture of kinship practices in an Early Neolithic tomb. Nature.

[18] Dabney J., Meyer M., Pääbo S. (2013). Ancient DNA damage. Cold Spring Harb. Perspect. Biol..

[19] Briggs A.W., Stenzel U., Johnson P.L., Green R.E., Kelso J., Prüfer K., Meyer M., Krause J., Ronan M.T., Lachmann M. (2007). Patterns of damage in genomic DNA sequences from a Neandertal. Proc. Natl. Acad. Sci. USA.

[20] Peyrégne S., Prüfer K. (2020). Present-Day DNA Contamination in Ancient DNA Datasets. Bioessays.

[21] Bendall K.E., Sykes B.C. (1995). Length heteroplasmy in the first hypervariable segment of the human mtDNA control region. Am. J. Hum. Genet..

[22] Stewart J.E., Fisher C.L., Aagaard P.J., Wilson M.R., Isenberg A.R., Polanskey D., Pokorak E., DiZinno J.A., Budowle B. (2001). Length variation in HV2 of the human mitochondrial DNA control region. J. Forensic Sci..

[23] Jónsson H., Ginolhac A., Schubert M., Johnson P.L., Orlando L. (2013). Map Damage 2.0: Fast approximate Bayesian estimates of ancient DNA damage parameters. Bioinformatics.

[24] Renaud G., Slon V., Duggan A.T., Kelso J. (2015). Schmutzi: Estimation of contamination and endogenous mitochondrial consensus calling for ancient DNA. Genome Biol..

[25] Neukamm J., Peltzer A., Nieselt K. (2021). Damage Profiler: Fast damage pattern calculation for ancient DNA. Bioinformatics.

[26] Nakatsuka N., Harney É., Mallick S., Mah M., Patterson N., Reich D. (2020). ContamLD: Estimation of ancient nuclear DNA contamination using breakdown of linkage disequilibrium. Genome Biol..

[27] Peyrégne S., Peter B.M. (2020). AuthentiCT: A model of ancient DNA damage to estimate the proportion of present-day DNA contamination. Genome Biol..

[28] Korneliussen T.S., Albrechtsen A., Nielsen R. (2014). ANGSD: Analysis of next generation sequencing data. BMC Bioinform..

[29] Feldman M., Fernández-Domínguez E., Reynolds L., Baird D., Pearson J., Hershkovitz I., May H., Goring-Morris N., Benz M., Gresky J. (2019). Late Pleistocene human genome suggests a local origin for the first farmers of central Anatolia. Nat. Commun..

[30] Skourtanioti E., Erdal Y.S., Frangipane M., Restelli F.B., Yener K.A., Pinnock F., Matthiae P., Özbal R., Schoop U., Guliyev F. (2020). Genomic history of neolithic to bronze age Anatolia, northern Levant, and southern Caucasus. Cell.

[31] Weissensteiner H., Pacher D., Kloss-Brandstätter A., Forer L., Specht G., Bandelt H., Kronenberg F., Salas A., Schönherr S. (2016). HaploGrep 2: Mitochondrial haplogroup classification in the era of high-throughput sequencing. Nucleic. Acids. Res..

[32] Van Oven M., Kayser M. (2009). Updated comprehensive phylogenetic tree of global human mitochondrial DNA variation. Hum. Mutat..

[33] Feldman M., Master D.M., Bianco R.A., Burri M., Stockhammer P.W., Mittnik A., Aja A.J., Jeong C., Krause J. (2019). Ancient DNA sheds light on the genetic origins of early Iron Age Philistines. Sci. Adv..

[34] Furtwängler A., Rohrlach A.B., Lamnidis T.C., Papac L., Neumann G.U., Siebke I., Reiter E., Steuri N., Hald J., Denaire A. (2020). Ancient genomes reveal social and genetic structure of Late Neolithic Switzerland. Nat. Commun..

[35] Zhang F., Ning C., Scott A., Fu Q., Bjørn R., Li W., Wei D., Wang W., Fan L., Abuduresule I. (2021). The genomic origins of the Bronze Age Tarim Basin mummies. Nature.

[36] Chyleński M., Ehler E., Somel M., Yaka R., Krzewińska M., Dabert M., Juras A., Marciniak A. (2019). Ancient mitochondrial genomes reveal the absence of maternal kinship in the burials of Çatalhöyük people and their genetic affinities. Genes.

[37] Brandt G., Haak W., Adler C.J., Roth C., Szécsényi-Nagy A., Karimnia S., Möller-Rieker S., Meller H., Ganslmeier R., Friederich S. (2013). Ancient DNA reveals key stages in the formation of central European mitochondrial genetic diversity. Science.

[38] Szécsényi-Nagy A., Brandt G., Haak W., Keerl V., Jakucs J., Möller-Rieker S., Köhler K., Mende B.G., Oross K., Marton T. (2015). Tracing the genetic origin of Europe’s first farmers reveals insights into their social organization. Proc. R. Soc. B Biol. Sci..

[39] Olalde I., Brace S., Allentoft M.E., Armit I., Kristiansen K., Booth T., Rohland N., Mallick S., Szécsényi-Nagy A., Mittnik A. (2018). The Beaker phenomenon and the genomic transformation of northwest Europe. Nature.

[40] Haak W., Lazaridis I., Patterson N., Rohland N., Mallick S., Llamas B., Brandt G., Nordenfelt S., Harney E., Stewardson K. (2015). Massive migration from the steppe was a source for Indo-European languages in Europe. Nature.

[41] Fu Q., Posth C., Hajdinjak M., Petr M., Mallick S., Fernandes D., Furtwängler A., Haak W., Meyer M., Mittnik A. (2016). The genetic history of ice age Europe. Nature.

[42] Lazaridis I., Nadel D., Rollefson G., Merrett D.C., Rohland N., Mallick S., Fernandes D., Novak M., Gamarra B., Sirak K. (2016). Genomic insights into the origin of farming in the ancient Near East. Nature.

[43] Wang C., Yeh H., Popov A.N., Zhang H., Matsumura H., Sirak K., Cheronet O., Kovalev A., Rohland N., Kim A.M. (2021). Genomic insights into the formation of human populations in East Asia. Nature.

[44] Hofmanová Z., Kreutzer S., Hellenthal G., Sell C., Diekmann Y., Díez-del-Molino D., Van Dorp L., López S., Kousathanas A., Link V. (2016). Early farmers from across Europe directly descended from Neolithic Aegeans. Proc. Natl. Acad. Sci. USA.

[45] Schuenemann V.J., Peltzer A., Welte B., Van Pelt W.P., Molak M., Wang C., Furtwängler A., Urban C., Reiter E., Nieselt K. (2017). Ancient Egyptian mummy genomes suggest an increase of Sub-Saharan African ancestry in post-Roman periods. Nat. Commun..

[46] Juras A., Chyleński M., Ehler E., Malmström H., Żurkiewicz D., Włodarczak P., Wilk S., Peška J., Fojtík P., Králík M. (2018). Mitochondrial genomes reveal an east to west cline of steppe ancestry in Corded Ware populations. Sci. Rep..

[47] Ning C., Zheng H., Zhang F., Wu S., Li C., Zhao Y., Xu Y., Wei D., Wu Y., Gao S. (2021). Ancient Mitochondrial Genomes Reveal Extensive Genetic Influence of the Steppe Pastoralists in Western Xinjiang. Front. Genet..

[48] Li H., Durbin R. (2009). Fast and accurate short read alignment with Burrows–Wheeler transform. Bioinformatics.

[49] Peltzer A., Jäger G., Herbig A., Seitz A., Kniep C., Krause J., Nieselt K. (2016). EAGER: Efficient ancient genome reconstruction. Genome Biol..

[50] Ewing B., Green P. (1998). Base-calling of automated sequencer traces using phred. II. Error probabilities. Genome Res..

[51] Ewing B., Hillier L., Wendl M.C., Green P. (1998). Base-calling of automated sequencer traces usingPhred. I. Accuracy assessment. Genome Res..

[52] Schubert M., Ginolhac A., Lindgreen S., Thompson J.F., Al-Rasheid K.A., Willerslev E., Krogh A., Orlando L. (2012). Improving ancient DNA read mapping against modern reference genomes. BMC Genom..

