# Supplementary material for: Mitochondrial DNA Consensus Calling and Quality Filtering for Constructing Ancient Human Mitogenomes: Comparison of Two Widely Applied Methods
Source: Int J Mol Sci. 2022 Apr 22;23(9):4651. doi: 10.3390/ijms23094651 (PMC9104972; doi:10.3390/ijms23094651)
Supplement: Supplementary file 1 [file ijms-23-04651-s001.zip › Figure S1 Haplogroup prediction accuracy for analyzed samples.pdf]

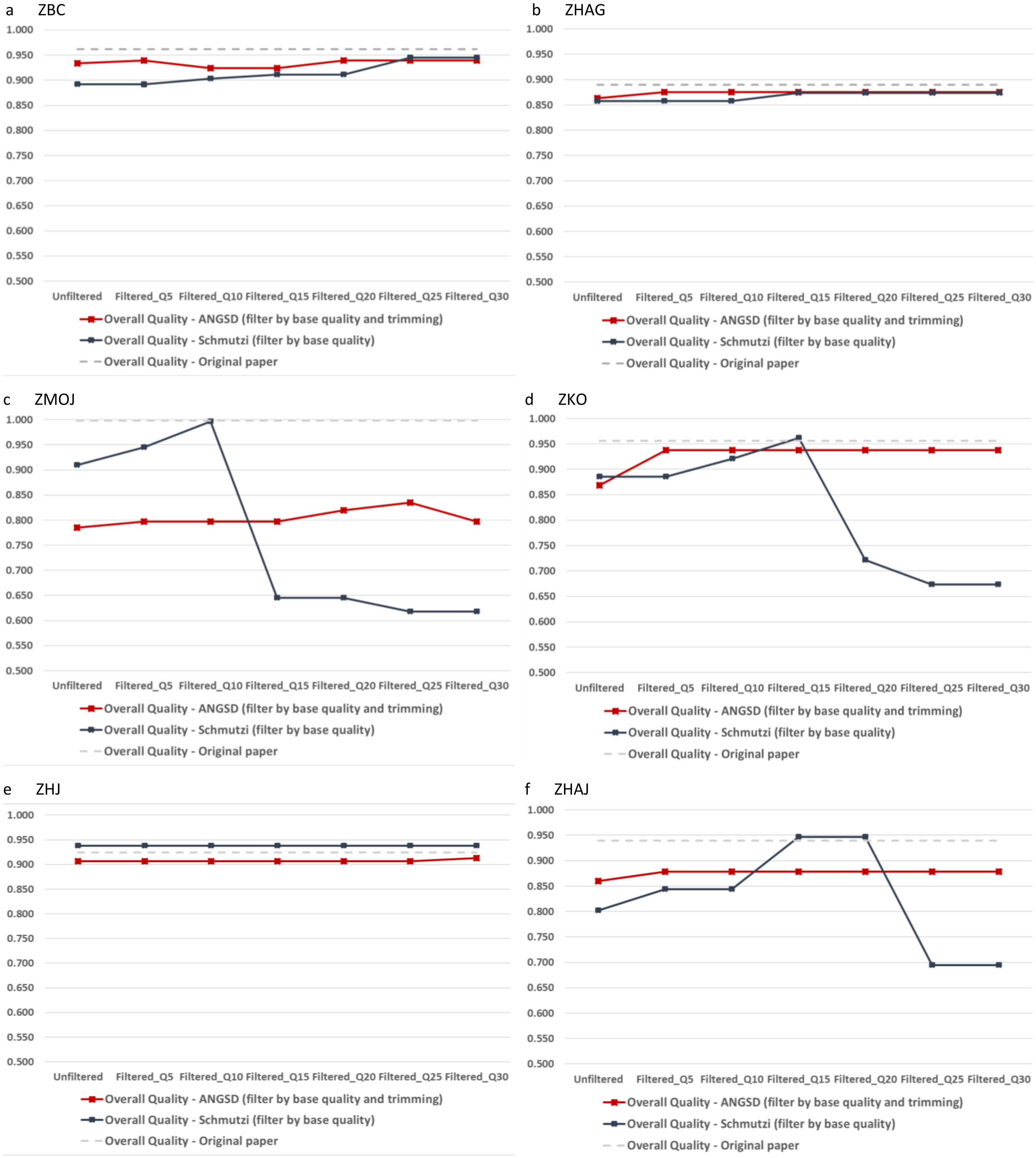
a ZBC
b ZHAG
c ZMOJ
d ZKO
e ZHJ
f ZHAJ
1.000
0.950
0.900
0.850
0.800
0.750
0.700
0.650
0.600
0.550
0.500
Unfiltered
Filtered_Q5
Filtered_Q10
Filtered_Q15
Filtered_Q20
Filtered_Q25
Filtered_Q30
Overall Quality - ANGSD (filter by base quality and trimming)
Overall Quality - Schmutzi (filter by base quality)
Overall Quality - Original paper

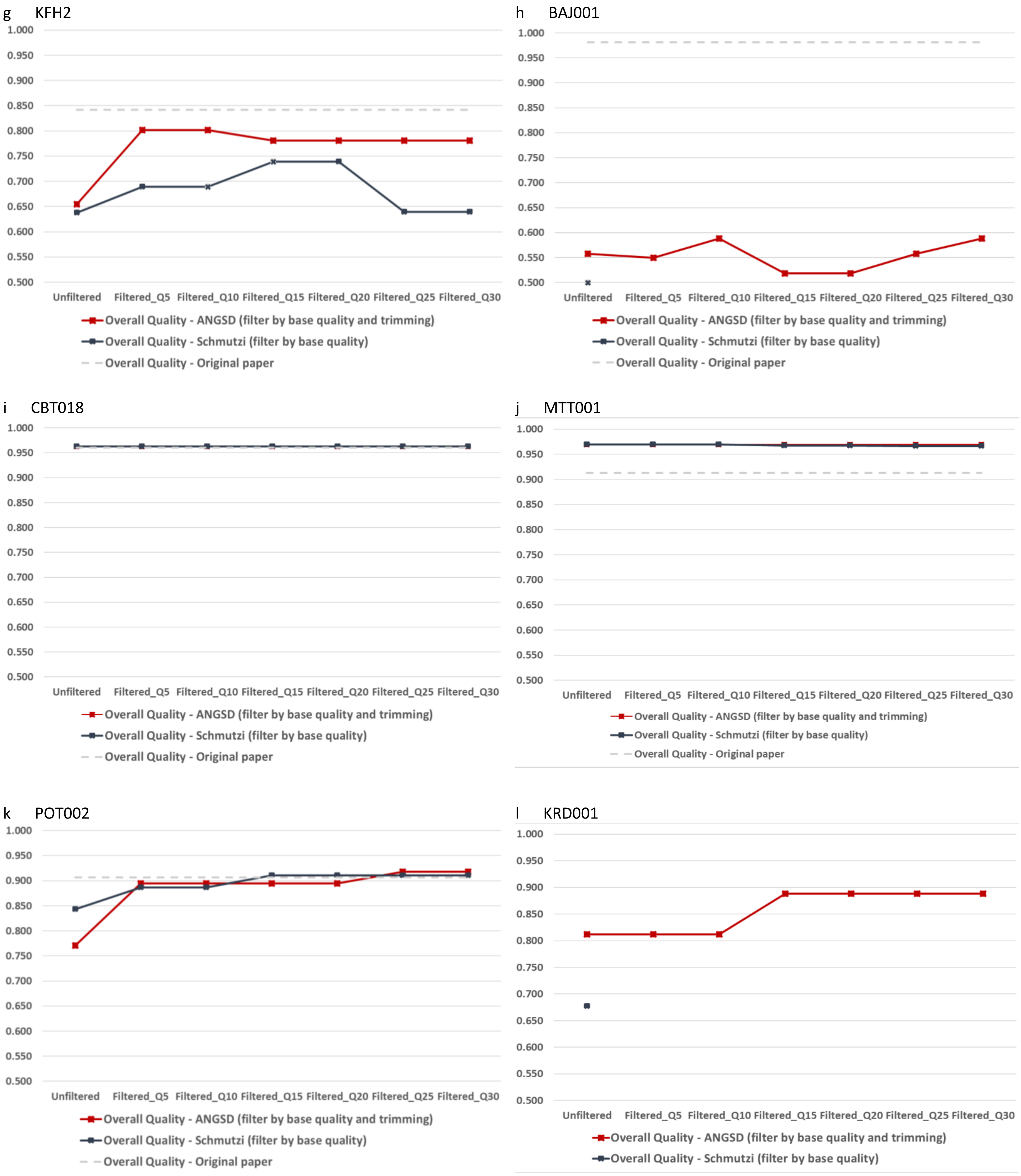

g KFH2
h BAJ001
i CBT018
j MTT001
k POT002
l KRD001
1.000
0.950
0.900
0.850
0.800
0.750
0.700
0.650
0.600
0.550
0.500
Unfiltered
Filtered_Q5
Filtered_Q10
Filtered_Q15
Filtered_Q20
Filtered_Q25
Filtered_Q30
Overall Quality - ANGSD (filter by base quality and trimming)
Overall Quality - Schmutzi (filter by base quality)
Overall Quality - Original paper

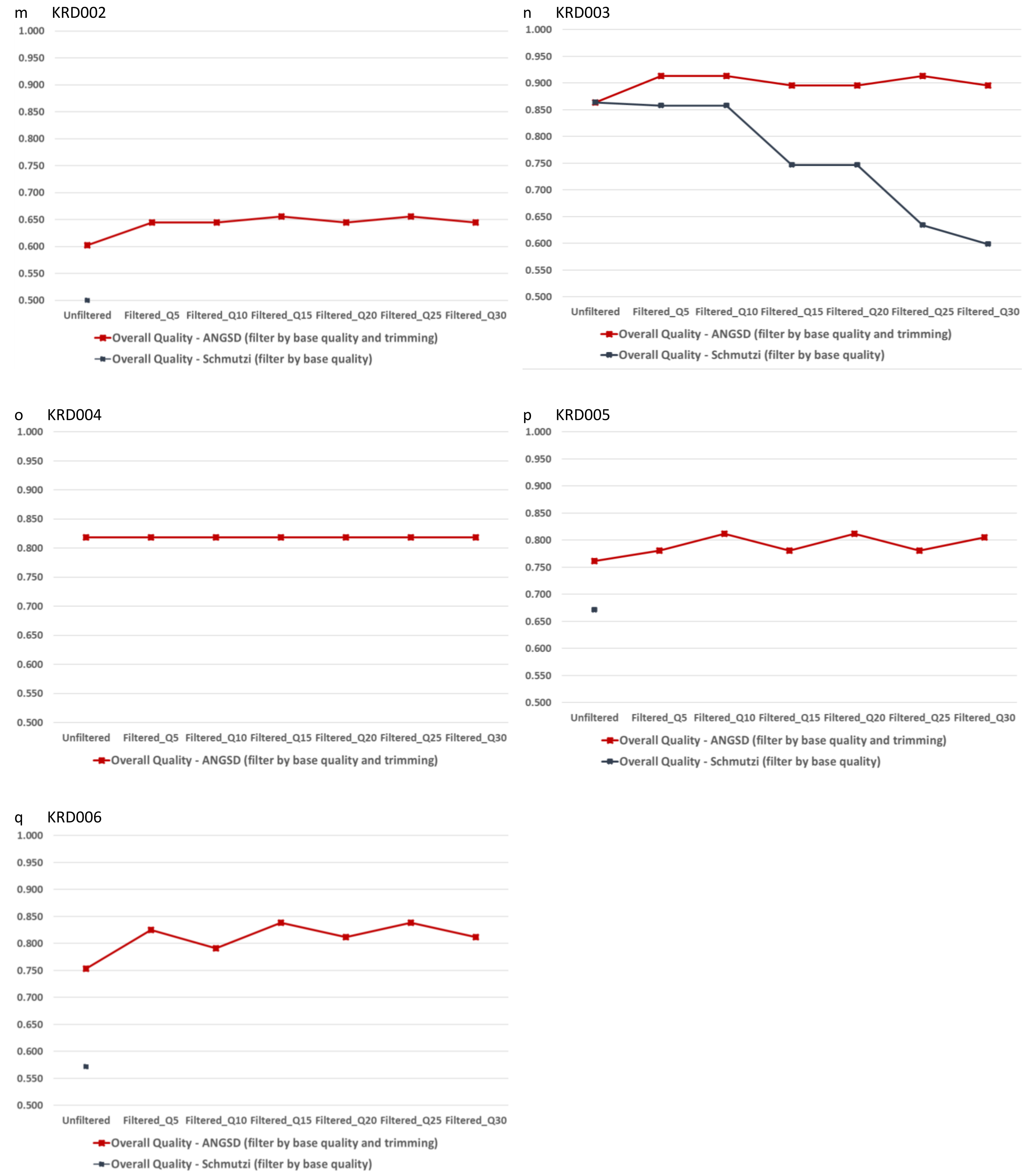

Figure S1. Haplogroup prediction accuracy for analyzed samples using mitogenome consensus sequence extracted from whole genome data, as derived by two alternative tools and pipelines (ANGSD *vs* schmutzi). Samples for which a quality curve is missing for one of the two tools, indicates that the approach did not result in a consensus mitogenome sequence, particularly after filtering: (**a**) sample ZBC; (**b**) sample ZHAG [29]; (**c**) sample ZMOJ [29]; (**d**) sample ZKO [29]; (**e**) sample ZHJ [29]; (**f**) sample ZHAJ [29]; (g) sample KFH2 [29]; (**h**) sample BAJ001 [29]; (**i**) sample CBT018 [30]; (**j**) sample MTT001 [30]; (**k**) sample POT002 [30]; (**l**) sample KRD001 [30]; (**m**) sample KRD002 [30]; (n) sample KRD003 [30]; (**o**) sample KRD004 [30]; (**p**) sample KRD005 [30];. (**q**) sample KRD006 [30].
