# Supplementary material for: Mitochondrial DNA Consensus Calling and Quality Filtering for Constructing Ancient Human Mitogenomes: Comparison of Two Widely Applied Methods
Source: Int J Mol Sci. 2022 Apr 22;23(9):4651. doi: 10.3390/ijms23094651 (PMC9104972; doi:10.3390/ijms23094651)
Supplement: Supplementary file 1 [file ijms-23-04651-s001.zip › Figure S2 Possible errors in mitochondrial consensus sequence calling for analyzed samples.pdf]

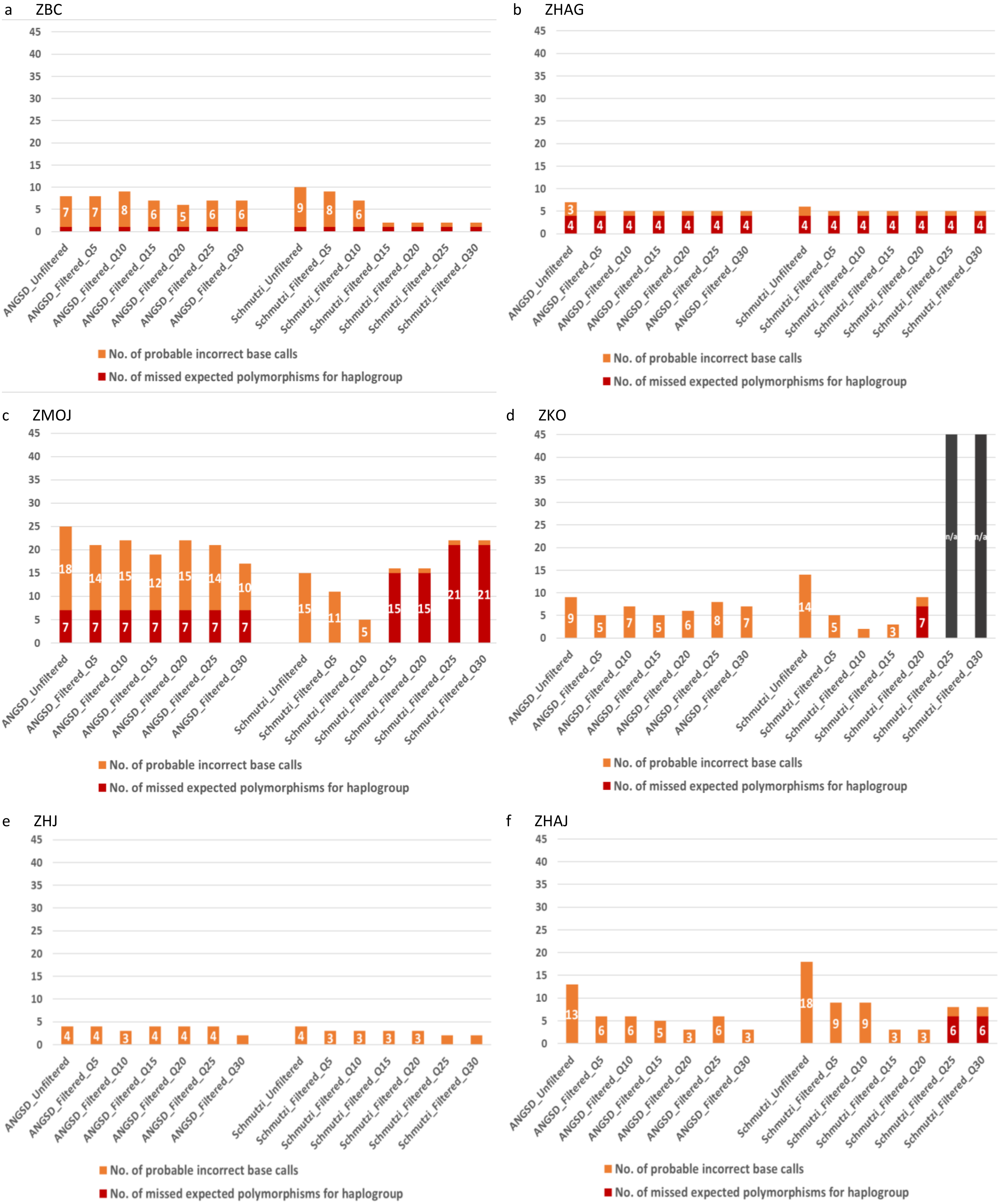
a ZBC
b ZHAG
c ZMOJ
d ZKO
e ZHJ
f ZHAJ
45
40
35
30
25
20
15
10
5
0
ANGSD_Unfiltered
ANGSD_Filtered_Q5
ANGSD_Filtered_Q10
ANGSD_Filtered_Q15
ANGSD_Filtered_Q20
ANGSD_Filtered_Q25
ANGSD_Filtered_Q30
Schmutzi_Unfiltered
Schmutzi_Filtered_Q5
Schmutzi_Filtered_Q10
Schmutzi_Filtered_Q15
Schmutzi_Filtered_Q20
Schmutzi_Filtered_Q25
Schmutzi_Filtered_Q30
No. of probable incorrect base calls
No. of missed expected polymorphisms for haplogroup
n/a

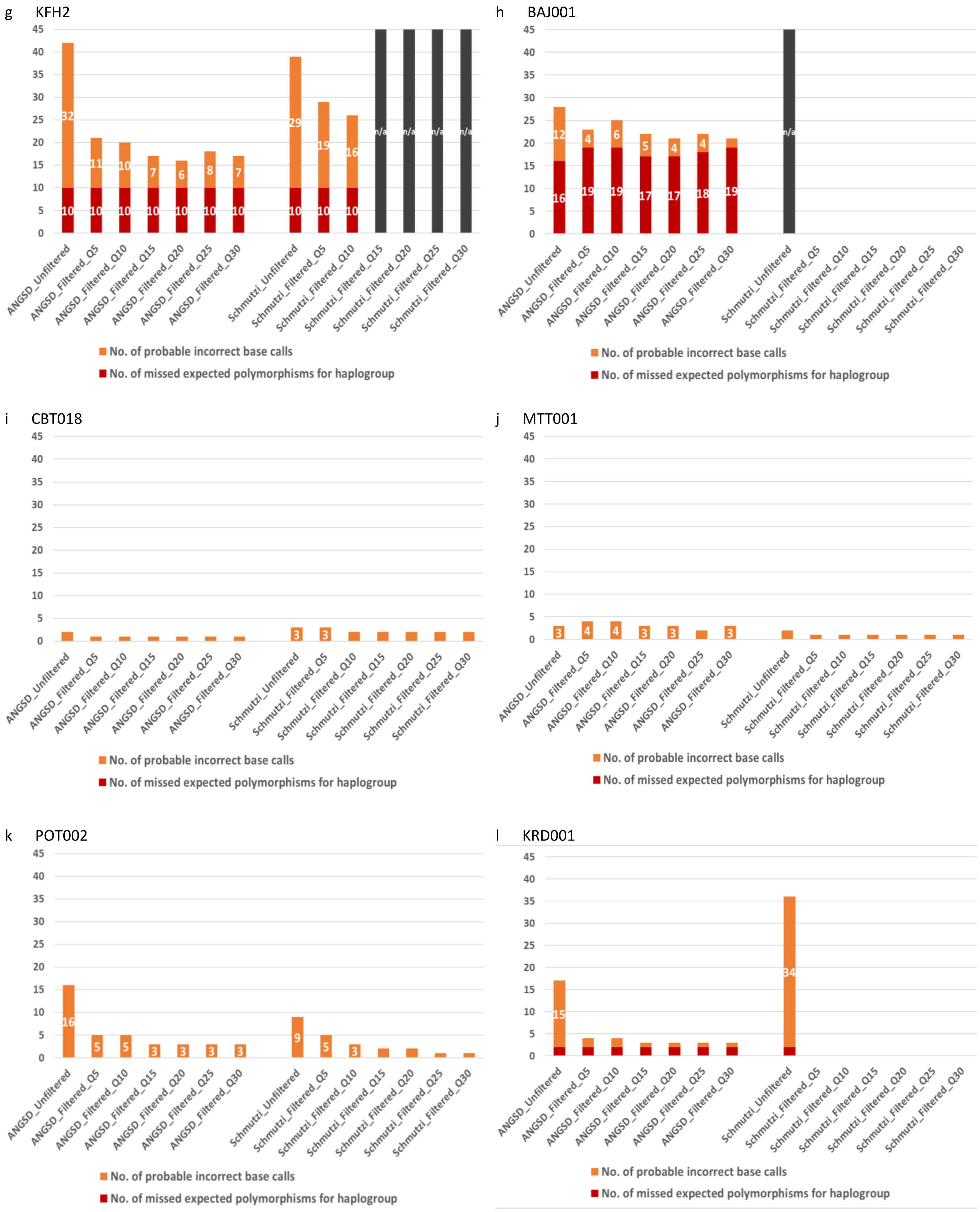
g KFH2
h BAJ001
i CBT018
j MTT001
k POT002
l KRD001
0
5
10
15
20
25
30
35
40
45
ANGSD_Unfiltered
ANGSD_Filtered_Q5
ANGSD_Filtered_Q10
ANGSD_Filtered_Q15
ANGSD_Filtered_Q20
ANGSD_Filtered_Q25
ANGSD_Filtered_Q30
Schmutzi_Unfiltered
Schmutzi_Filtered_Q5
Schmutzi_Filtered_Q10
Schmutzi_Filtered_Q15
Schmutzi_Filtered_Q20
Schmutzi_Filtered_Q25
Schmutzi_Filtered_Q30
No. of probable incorrect base calls
No. of missed expected polymorphisms for haplogroup
32
11
10
7
6
8
7
29
19
16
10
n/a
12
4
6
5
4
4
16
19
19
17
17
18
19
n/a
3
3
3
4
4
3
3
3
16
5
5
3
3
3
3
9
5
3
15
34

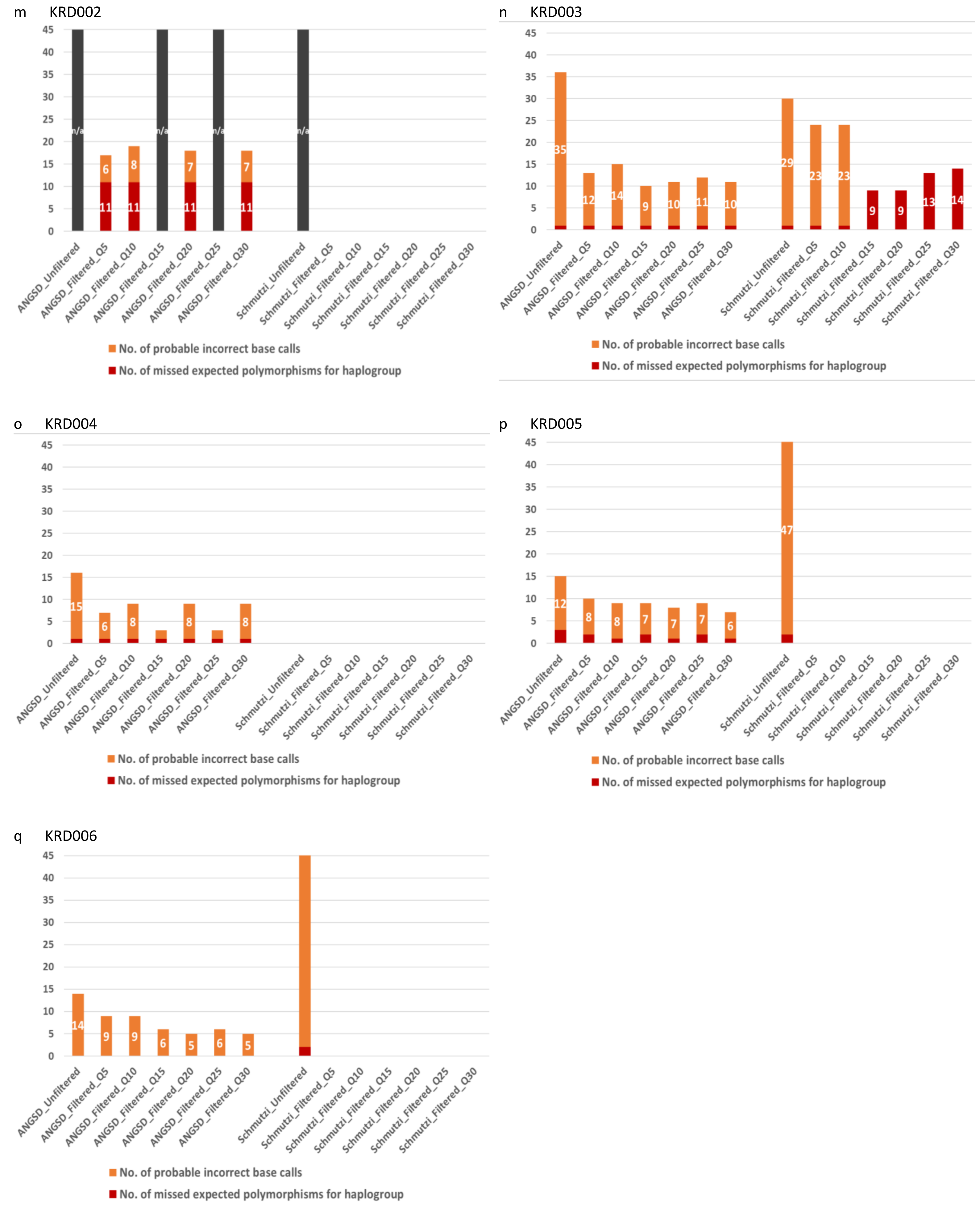

Figure S2. Possible errors in mitochondrial consensus sequence calling for analyzed samples as indicated during haplogroup prediction, presented by base quality filter, by two alternative tools and pipelines (ANGSD *vs* schmutzi). Dark grey bars indicate that there was a wrong haplogroup perdition, thus the number of missed expected polymorphisms and possible incorrect base calls is not applicable. These haplotypes should be assumed to have the maximum number of errors in both parameters. Samples for which a bar is missing, indicates that the approach did not result in a consensus mitogenome sequence, particularly after filtering: (a) sample ZBC [29]; (b) sample ZHAG [29]; (c) sample ZMOJ [29]; (d) sample ZKO [29]; (e) sample ZHJ [29]; (f) sample ZHAJ [29]; (g) sample KFH2 [29]; (h) sample BAJ001 [29]; (i) sample CBT018 [30]; (j) sample MTT001 [30]; (k) sample POT002 [30]; (l) sample KRD001 [30]; (m) sample KRD002 [30]; (n) sample KRD003 [30]; (o) sample KRD004 [30]; (p) sample KRD005 [30];. (q) sample KRD006 [30].
