# Supplementary material for: Mitochondrial DNA Consensus Calling and Quality Filtering for Constructing Ancient Human Mitogenomes: Comparison of Two Widely Applied Methods
Source: Int J Mol Sci. 2022 Apr 22;23(9):4651. doi: 10.3390/ijms23094651 (PMC9104972; doi:10.3390/ijms23094651)
Supplement: Supplementary file 1 [file ijms-23-04651-s001.zip › Figure S3 Association between different sample characteristics and derived sequence quality.pdf]

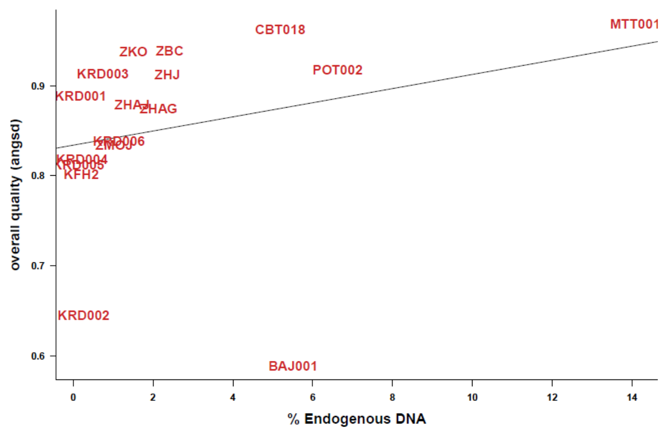
overall quality (angsd)
% Endogenous DNA
0.6
0.7
0.8
0.9
0
2
4
6
8
10
12
14
MTT001
CBT018
ZKO
ZBC
KRD003
ZHJ
POT002
KRD001
ZHAG
KFH2
KRD002
BAJ001

(a)

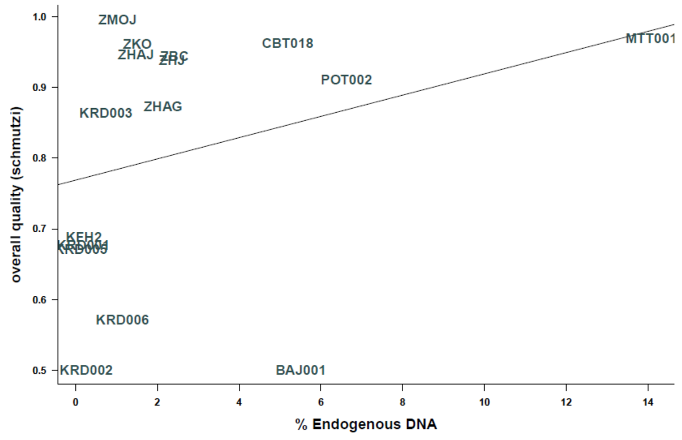
overall quality (schmutzi)
% Endogenous DNA
0.5
0.6
0.7
0.8
0.9
1.0
0
2
4
6
8
10
12
14
ZMOJ
ZKO
ZHAJ
CBT018
MTT001
POT002
KRD003
ZHAG
KFH2
KRD006
KRD002
BAJ001

(b)

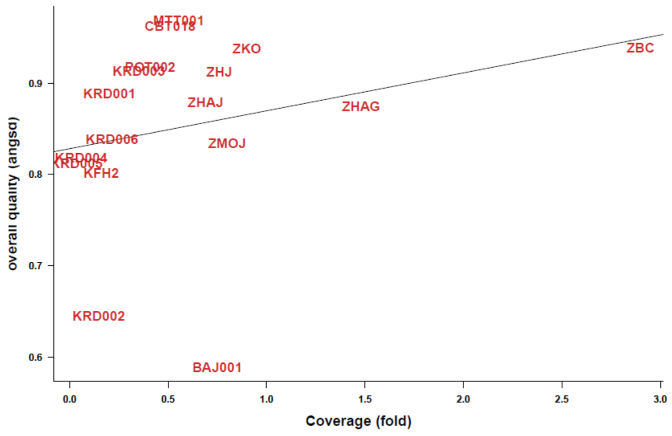
overall quality (angsd)
Coverage (fold)
0.6
0.7
0.8
0.9
0.0
0.5
1.0
1.5
2.0
2.5
3.0
MTT001
ZKO
ZBC
ZHJ
KRD001
ZHAJ
ZHAG
KRD006
ZMOJ
KFH2
KRD002
BAJ001

(c)

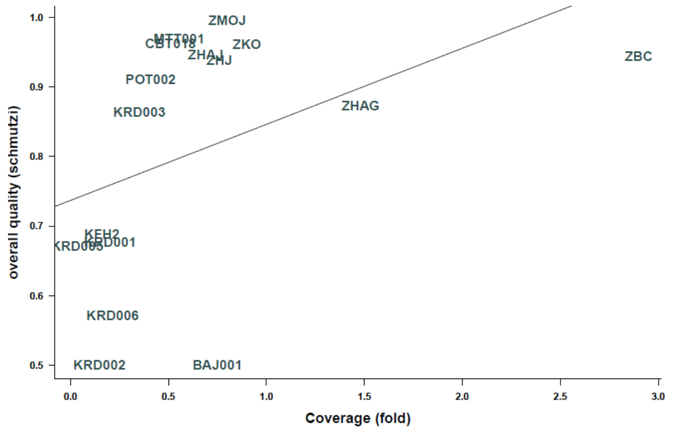
overall quality (schmutzi)
Coverage (fold)
0.5
0.6
0.7
0.8
0.9
1.0
0.0
0.5
1.0
1.5
2.0
2.5
3.0
ZMOJ
ZKO
ZBC
ZHJ
POT002
KRD003
ZHAG
KFH2
KRD006
KRD002
BAJ001

(d)

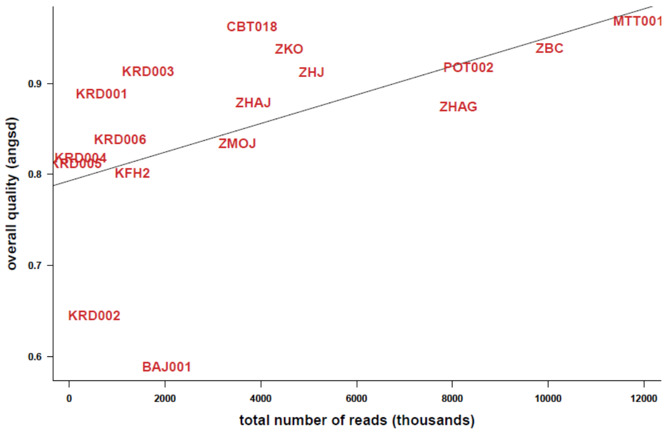
overall quality (angsd)
total number of reads (thousands)
0.6
0.7
0.8
0.9
0
2000
4000
6000
8000
10000
12000
CBT018
MTT001
ZKO
ZBC
KRD003
ZHJ
POT002
KRD001
ZHAJ
ZHAG
KRD006
ZMOJ
KRD004
KFH2
KRD002
BAJ001

(e)

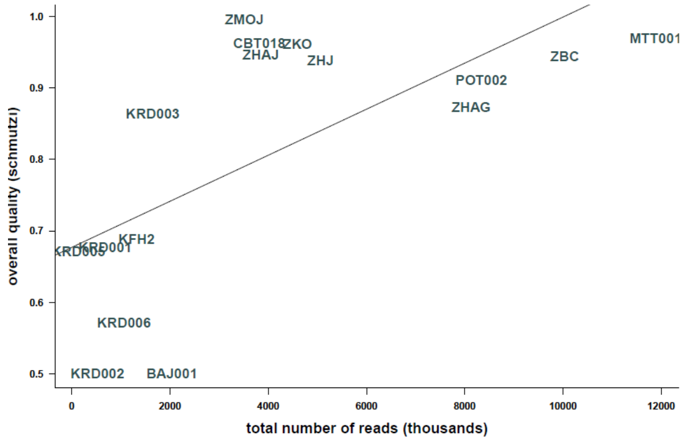
overall quality (schmutzi)
total number of reads (thousands)
0.5
0.6
0.7
0.8
0.9
1.0
0
2000
4000
6000
8000
10000
12000
ZMOJ
ZKO
ZHAJ
ZHJ
MTT001
ZBC
POT002
ZHAG
KRD003
KFH2
KRD006
KRD002
BAJ001

(f)

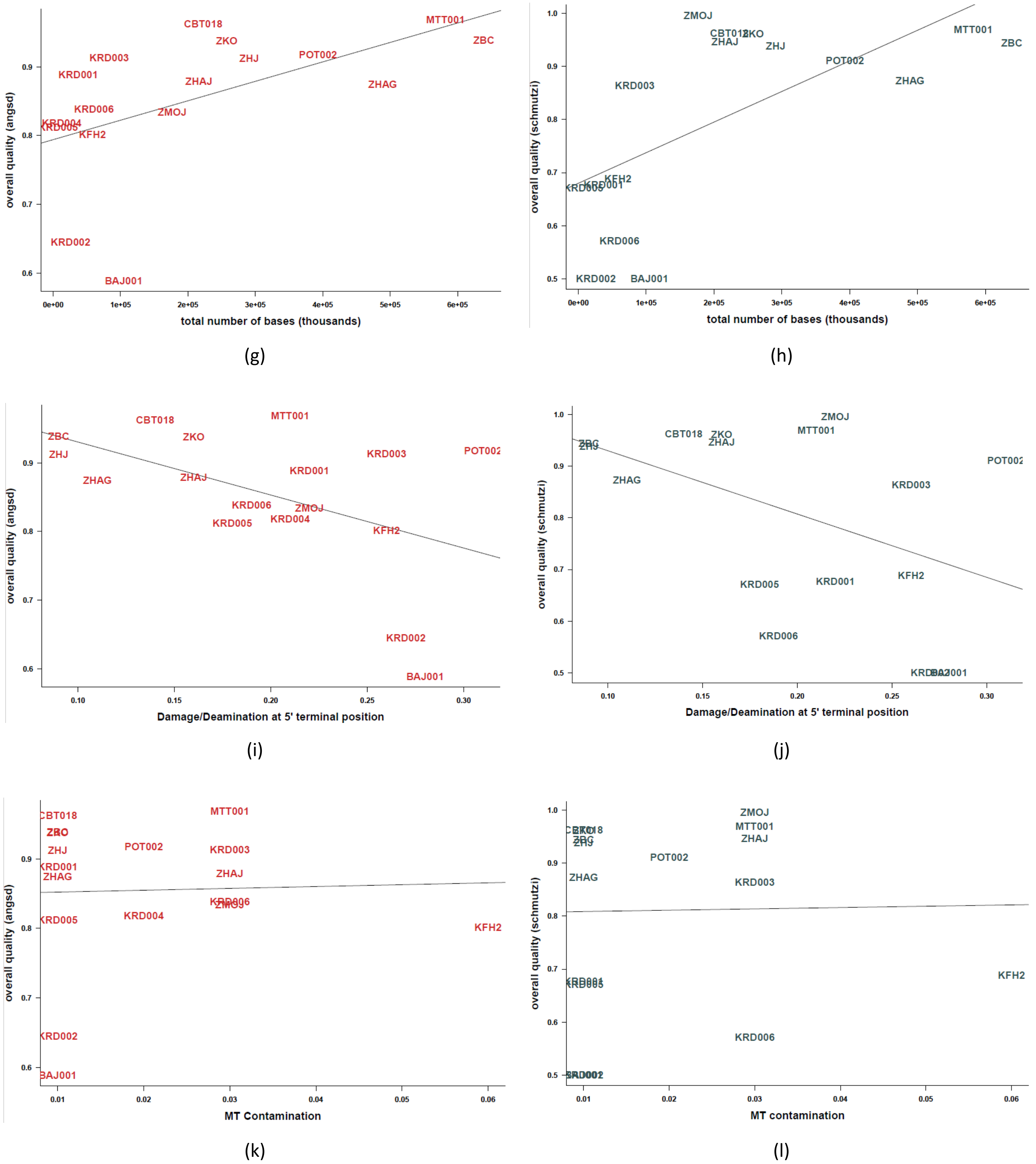

Figure S3. Association between different sample characteristics and derived sequence quality, by two alternative tools and pipelines (ANGSD *vs* schmutzi): (**a**) Proportional endogenous DNA *vs* overall ANGSD-derived sequence quality; (**b**) Proportional endogenous DNA *vs* overall schmutzi-derived sequence quality; (**c**) Coverage *vs* overall ANGSD-derived sequence quality; (**d**) Coverage *vs* overall schmutzi-derived sequence quality; (**e**) Total number of reads *vs* overall ANGSD-derived sequence quality; (**f**) Total number of reads *vs* overall schmutzi-derived sequence quality; (g) Total number of bases *vs* overall ANGSD-derived sequence quality; (**h**) Total number of bases *vs* overall schmutzi-derived sequence quality; (**i**) Damage at 5' position *vs* overall ANGSD-derived sequence quality; (**j**) Damage at 5' position *vs* overall schmutzi-derived sequence quality; (**k**) MT contamination *vs* overall ANGSD-derived sequence quality; (**l**) MT contamination *vs* overall schmutzi-derived sequence quality.
