# Supplementary figures and images for: Mitochondrial DNA Consensus Calling and Quality Filtering for Constructing Ancient Human Mitogenomes: Comparison of Two Widely Applied Methods
Source: Int J Mol Sci. 2022 Apr 22;23(9):4651. doi: 10.3390/ijms23094651 (PMC9104972; doi:10.3390/ijms23094651)

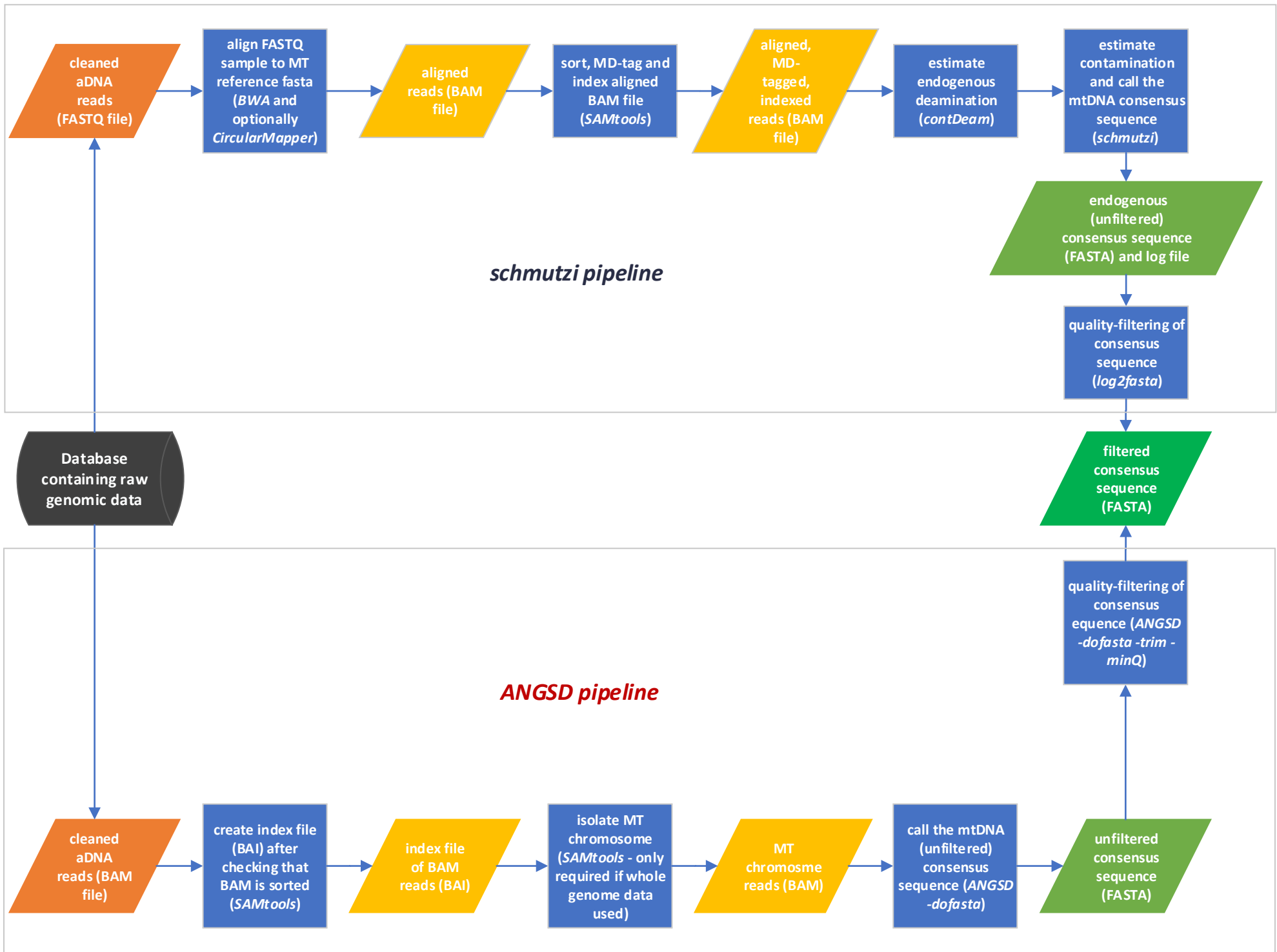

Supplement: Supplementary file 1 [file ijms-23-04651-s001.zip › Figure S4 Flowchart of pipelines from raw genomic data to sequence fasta.pdf]
